# A Review on the Synthesis, Properties, and Utilities of Functionalized Carbon Nanoparticles for Polymer Nanocomposites

**DOI:** 10.3390/polym13203547

**Published:** 2021-10-14

**Authors:** Jun-Ven Lim, Soo-Tueen Bee, Lee Tin Sin, Chantara Thevy Ratnam, Zuratul Ain Abdul Hamid

**Affiliations:** 1 Department of Mechanical and Material Engineering, Lee Kong Chian Faculty of Engineering and Science, Universiti Tunku Abdul Rahman, Bandar Sungai Long, Kajang 43000, Selangor, Malaysia; junvenlim@1utar.m; 2 Department of Chemical Engineering, Lee Kong Chian Faculty of Engineering and Science, Universiti Tunku Abdul Rahman, Bandar Sungai Long, Kajang 43000, Selangor, Malaysia; 3 Radiation Processing Technology Division Malaysian Nuclear Agency, Bangi, Kajang 43000, Selangor, Malaysia; Chantara@nuclearmalaysia.gov.my; 4 School of Materials and Mineral Resources Engineering, Engineering Campus, Universiti Sains Malaysia, Nibong Tebal 14300, Pulau Pinang, Malaysia; srzuratulain@usm.my

**Keywords:** carbon nanoparticles, functionalization, polymer nanocomposites, mechanical properties

## Abstract

Carbon can form different allotropes due to its tetravalency. Different forms of carbon such as carbon nanotubes (CNTs), carbon nanofibers, graphene, fullerenes, and carbon black can be used as nanofillers in order to enhance the properties of polymer nanocomposites. These carbon nanomaterials are of interest in nanocomposites research and other applications due to their excellent properties, such as high Young’s Modulus, tensile strength, electrical conductivity, and specific surface area. However, there are some flaws that can be found in the carbon nanoparticles such as tendency to agglomerate, insoluble in aqueous or organic solvents or being unreactive with the polymer surface. In this study, the aim is to study functionalization in order to rectify some of these shortcomings by attaching different functional groups or particles to the surface of these carbon nanoparticles; this also enables the synthesis of high-performance polymer nanocomposites. The main findings include the effects of functionalization on carbon nanoparticles and the applications of polymer nanocomposites with carbon nanoparticles as nanofillers in the industry. Additionally, the different methods used to produce polymer composites such as in situ polymerization, solution mixing and melt blending are studied, as these methods involve the dispersion of carbon nanofillers within the polymer matrix.

## 1. Introduction

Carbon, the sixth element in the periodic table, is a nonmetallic chemical element that is tetravalent, which means it has four valence electrons that it can form four covalent bonds with. This valency of carbon gives rise to its excellent catenation ability, where it is able to form chemical bonds with other carbon atoms or elements which give rise to molecules that are essential to organic chemistry and life itself. Since atomic carbon is not very stable, carbon atoms will arrange themselves into different structural forms in an attempt to stabilize themselves, which are known as allotropes. The more well-known allotropes of carbon are diamond and graphite, although other allotropes of carbon have been discovered in recent decades such as fullerenes and graphene [1]. Different forms of carbon have also been synthesized by humans, such as synthetic diamond, carbon black, graphitic fibers, carbon nanotubes (CNTs), glassy carbon, and coke, which have many industrial applications [2]. These different forms and structures can arise due to the special hybridization properties of carbon. Carbon is able to form sp, sp^2^ and sp^3^ hybrid orbitals. The sp hybridization will form a linear structure in carbon, while sp^2^ hybridization gives rise to planar structure and sp^3^ hybridization results in a tetrahedral geometry.

Additionally, many of these carbon nanoparticles possess unique and useful attributes, for instance, graphene has excellent mechanical, electrical and thermal properties due to the long-range π conjugation between its carbon atoms [3]. These properties make carbon nanomaterials desired for a wide array of technological applications. This is seen in recent nanocomposites research, where many studies have been dedicated towards the use of carbon nanoparticles as nanofillers and the effect these nanofillers have on polymer composites. Regrettably, the use of these carbon based nanofillers in nanocomposites is limited by their expensive production cost. For example, currently in 2021, the price of producing graphene costs approximately USD 100/g [4]. However, the good news is that technology can aid in reducing the price of carbon nanomaterials, such as new breakthroughs in the synthesis process. This can be seen in the reduction of the price from 2010, where graphene the size of a stamp would cost tens of thousands of dollars to make. It is believed with the rise of newer technologies the price would further decline.

In this review paper, the development of polymer/carbon nanoparticles composite research will be discussed, which includes studies on the different types of carbon nanoparticles, the functionalization of carbon nanomaterials and the effect on its mechanical and electrical properties, the different preparation methods of polymer nanocomposites and the use of polymer nanocomposites in various industries.

Polymers are materials that consist of very large molecules that are built up by the repetitive bonding together of monomers. Polymers play a big part in the engineering industry due to its many benefits such as cheapness, ease of processing, and possible recyclability compared to other substances [5]. Certain polymers, however, possess unsatisfactory properties such as low tensile strength and low electrical conductivity. The research into polymer nanocomposites could improve these unsatisfactory attributes as it is able to produce high performance materials able to be applied in harsh environments out of these feeble polymers. Polymer nanocomposites are described as polymers that have nanoparticles or nanofillers incorporated in its matrix [6]. The nanofillers can be one dimensional (carbon fibers), two dimensional (graphite nanosheets) or three dimensional (spherical particles), but for this review paper carbon-based nanofillers will be focused on fullerenes, CNTs, graphene, and graphene oxide (GO).

Research into polymer nanocomposites is important as only a small amount of nano-inorganic fillers are required to be mixed with the polymer before considerable improvement can be seen in the mechanical, electrical and thermal properties of the resulted composite. Typical filler amount of less than 5 wt% brings about effective improvement of the nanocomposite properties due to the large surface area to volume ratio of fillers [7]. This opens up new possibilities for polymers that have undesirable qualities to be reinvented as high performance nanocomposites.

Functionalization of carbon nanoparticles will be examined here as it is an effective way to disperse it in the polymer matrix and to enhance interface interaction with the polymer itself. Other lesser known forms of functionalization are also discussed in this review. For example, the effect of supercritical water treatment on carbon nanofillers is also a subject of growing interest in nanocomposites research due to its effect in preparing polymer composites. For example, the dispersion of the carbon nanosheets in polystyrene (PS) matrix is enhanced due to the ability of the supercritical water to activate various functional groups on the surface of the nanosheet [8]. It is also worth noting that supercritical water possesses many special properties such as owning excellent oxidation, solubility and diffusion abilities along with increased collision rates. However, there are only a few papers devoted to the investigation of the supercritical water treatment on carbon nanoparticles. This is because supercritical water has generally been used for decomposing toxic organic waste materials [9], while being neglected in its use in the polymer field. Other supercritical solvents that can also be used in functionalization or exfoliation of carbon nanomaterials include supercritical carbon dioxide and supercritical ethanol.

In summary, this review article will discuss the types of carbon nanoparticles, the various synthesis methods of the carbon nanomaterials, the types of functionalization for the nanoparticles, the effect on the properties after functionalization, the polymer nanocomposites with carbon nanoparticles as nanofillers that can be created and their use in the industry, along with the production methods for these polymer nanocomposites.

### Research Methodology

This is a review paper, and as such there are no experiments or research methodology used in order to obtain the results and facts required. However, it is still important to provide the methods we used so that it may provide a better understanding of the literature we selected. Since this is a review article, the main point of reference that is selected are other review papers under the same area of research, such as polymer nanocomposites, carbon nanoparticles, types of functionalization, and their effect in carbon-based nanofillers. One such example is “A review on carbon nanotube” by Rathinavel et al. [10], which provided an extensive look on the synthesis, functionalization and applications of CNTs.

Besides review articles, research articles mentioned in the review paper were also looked into, in order to both confirm the review paper’s validity and also obtain new information. Furthermore, websites such as ScienceDirect and ResearchGate offer access to many peer-reviewed research papers, which helps us to study new findings or get new insight that may have been overlooked. As such, we believe that our research methodology and findings are adequate.

## 2. Types of Carbon Nanoparticles

### 2.1. Carbon Nanosheets

Carbon or graphite nanosheets are carbon nanoparticles that are composed of a few to many layers of self-aligned graphene sheet [11]. The interlayer distance maintained due to the van der Waal forces between the carbon nanosheets is around 0.34 nm [12]. This two-dimensional nanosheet has a thickness and lateral dimension ranging from less than 100 nm; it also has an exceptionally high surface-to-volume ratio. They are a mechanically robust, free standing two-dimensional (2D) polymeric material made out of layers of hexagon-shaped lattice of sp^2^ hybridized carbon atoms [13]. The many layers of the carbon nanosheets are produced when the aromatic graphene monolayers cross-linked with each other with electrons. Due to these special characteristics, carbon nanosheets possess many interesting properties. For instance, the nanosheets show high thermal and electrical conductivity, high tensile strength and superior barrier properties [14]. Some example of the application of carbon nanosheets include being used as biosensors, energy storage devices, catalyst supports, and supercapacitors.

Furthermore, the surface of these nanosheets can be easily altered by increasing different functional groups on it, which makes the nanosheets good candidates for the production of new or unique polymer nanocomposites [15]. Incorporation of functional groups on the surface of the carbon nanosheets can also help enhance its characteristics such as increasing electrical conductivity, tensile strength and capacity.

Although recent efforts have been made to scale-up the production of graphene or modified graphene, only carbon nanosheets, which consist of stacked graphene layers bounded to each other by van der Waals forces, can currently be produced at the scales needed for use in composite materials and structural applications. However, it is still not possible to produce carbon nanosheets with specific size, shape or thickness. It is important to understand the different synthesis methods because the characteristics of the carbon nanosheets such as degree of graphitization, morphology or pore structures are influenced by the various synthesis methods. The precursors or starting materials can differ from graphite materials, polymers, resins, or tiny to large organic compounds.

### 2.2. Graphene

Graphene is a two-dimensional (2D) allotrope of carbon that is made out of a single layer of sp^2^-hybridized carbon atoms that is arranged in a hexagonal lattice. Graphene is also considered as a basic component of other graphite related materials such as CNTs, graphite oxide, and fullerene [16]. As a 2D nanostructure, graphene possesses a wide, open, flat layer with a high surface area, high Young’s Modulus and exceptionally high electrical conductivity. To expand on its properties: according to the research by Lee et al. [17], graphene is the strongest thing put to test, with its intrinsic tensile strength and Young’s Modulus being shown to be 130 GPa and 1.0 TPa, respectively, after being tested with an atomic force microscope (AFM). Balandin et al. [18] also report of an exceedingly high thermal conductivity of 5300 Wm^−1^ K^−1^, as compared to that of pyrolytic graphite (2000 Wm^−1^ K^−1^) or diamond (1000 Wm^−1^ K^−1^) at ambient conditions. As a result of these unique properties, more and more research interest can be seen in using graphene. It is also widely used as a nanofiller for producing nanocomposites as it can be easily dispersed in most polymer matrices.

To summarize its history: the existence of graphene was theorized for decades, although scientists at that time were unsure how to produce it. However, in 1962, Boehm and his colleagues managed to separate thin carbon foils from graphite oxide through heating and chemical reduction [19]. Before 2004, most scientists believed that monolayer graphene was too thermodynamically unstable to exist in the free state [20]. It was later in 2004 when Andre Geim and Konstantin Novoselov successfully isolated a single layer of graphene via mechanical exfoliation of graphite by using scotch tape [21].

It is a well-known fact that the unique properties of graphene are derived from its single layer. However, there are also disadvantages of graphene. For example, graphene sheets with high surface area have a tendency to aggregate with one another or even reform graphite through π-π stacking and van der Waals interactions [22]. The aggregation of graphene is seen as undesirable as their unique characteristics stem from the presence of individual sheets. Additionally, it will also hamper the ability of the graphene to be dispersed homogenously within the polymer matrix when it is used as a nanofiller, which will negatively affect the electrical and mechanical properties of the resultant polymer composite [23]. This ties in with one of the subjects of this review paper as aggregation can be decreased by incorporating various functional groups (functionalization) onto the graphene sheets. The addition of functional groups such as hydroxyl and carboxylic acid groups on the surface of graphene helps prevents the graphene sheets from aggregating due to their large size and the strong dipole–dipole intermolecular forces [24]. The incorporation of functional groups also helps to evenly disperse the graphene sheets in hydrophobic or hydrophobic solvent and in the polymer matrix. There are also other reasons why the use of pure graphene may not be suitable for certain nanotechnology. This can be seen when graphene based field effect transistors cannot be turned off effectively due to the absence of a band gap in pristine graphene [25].

### 2.3. Carbon Nanotubes

CNTs were first discovered in the year 1991, when Sumio Iijima grew them or the “graphitic carbon needles” as he called them, on the negative end of a carbon electrode which was used during a direct current arc evaporation of carbon in a vessel filled with argon gas. The CNTs Iijima produced ranged from 4 to 30 nm in diameter and reached a length of 1 μm. The number of walls of the nanotubes fabricated also differed from 2 to 50 sheets [26]. As can be gleaned from the results of Iijima’s experiment, CNTs are named after their size, with their tubes having diameters being a few nanometers. The structure of CNTs are made when one or more graphitic layers are folded to form a hollow tubule, which will result in a single-wall carbon nanotube (SWCNT) or a multi-wall carbon nanotube (MWCNT) [26]. The graphitic layers are composed of a 2D hexagon-shaped lattice of sp^2^ bonded carbon atoms. Additionally, CNTs can have electrical properties similar to a semiconductor or metal based on its diameter and the folding angle of the graphitic layer.

CNTs are one of the many carbon nanoparticles that are used in the field of nanotechnology. This is because of the unique mechanical, thermal and electrical properties of the nanotubes that allow researchers to create nanocomposites with many beneficial features fit for a large array of applications [27]. Some of the unique characteristics include having a high Young’s Modulus (1.0 TPa), exceptional tensile strength (50–250 GPa) and electrical conductivity between metallic and semi conductive [28]. Furthermore, they are a nanofiller with a tubular structure that has a diameter in the range of nanometers as well as a large aspect ratio (the ratio of its length to its width).

In brief, polymer nanocomposites made from both thermoplastics and thermosets being reinforced with CNTs have seen applications in the engineering field due to their ease in production, low cost and good mechanical properties. An example of a polymer composite using CNTs as nanofiller is the epoxy/CNT nanocomposite. The epoxy composites are used in aerospace engineering or the aircraft sector due to its excellent thermal resistance and high specific strength (strength to weight ratio) [29]. These composites are used in the wing-tip fairings of the Lockheed Martin F-35. The CNT reinforced epoxy composites are also used in Tomahawk missiles and aircrafts.

#### 2.3.1. Single-Wall Carbon Nanotubes

There are three types of CNTs, all of which are separated based on the number of inner walls within the CNTs. The first of which is the SWCNT. SWCNT consists of a single sheet of graphene that is rolled up to form a cylinder with diameters around the range of 0.4–3 nm. Catalysts are used during the chemical vapor deposition (CVD) process, which is an easy to control and efficient way to produce SWCNTs on an enormous scale with low cost yet with high product purity [30].

#### 2.3.2. Multi-Wall Carbon Nanotubes

Contrasting with the SWCNTs, MWCNTs contains multiple (concentrically interlinked) layers of rolled graphene. The structure of MWCNT can be split into two models: the Russian Doll model and Parchment model. For the Russian Doll model, the CNTs are composed of multiple graphitic layers arranged in concentric cylinders, where the diameter of the outer wall must be larger than the inner walls. Meanwhile, the Parchment Model resembles a scroll of parchment or a rolled up newspaper, with one layer of graphene rolled around itself. The Russian Doll model also appears more commonly in MWCNTs. Additionally, the interlayer distance in the MWCNTs is similar to the distance between the layers in graphite, which is around 0.34 nm.

#### 2.3.3. Double-Wall Carbon Nanotubes

**Figure 1:**
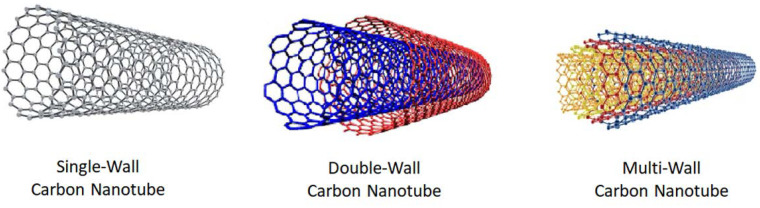
Types of carbon nanotubes.

Double-walled carbon nanotubes (DWCNTs) are nanotubes that consist of two CNTs where the smaller one is nested within the larger one. Similar to MWCNTs, the distance between the two nanotubes is approximately 0.34 nm, which is similar to the distance between two graphitic layers [31]. While the properties of DWCNT are comparable to SWCNT as their structure is similar to one another, Piao et al. [32] have reported that DWCNTs are more resistant to aggressive chemical attacks due to the protection provided by the outer wall. This result allows for the grafting of several functional groups on the surface of the outer wall (functionalization) in order to enhance the properties of the DWCNT without substantially altering its overall mechanical and electrical properties. This is because covalent functionalization of SWCNTs will generally break some of the carbon double bonds as their constituent atoms are exposed to the chemicals, resulting in “holes” in the nanotube. However, the functionalization of DWCNTs only takes place on the outer wall, which is proven when the inner nanotube is untouched during the diazonium functionalization by Piao et al. [32]. All three CNTs are illustrated in Figure 1.

#### 2.3.4. Basis of Chirality of Carbon Nanotubes

To understand chirality, we must first go back to graphene, as CNTs are nothing but rolled up tubes of graphene. To create a seamless CNT, it is in order that two hexagons in the graphene sheet overlap with each other. The chiral vector is the line connecting the center of the two hexagons, which also specifies the structure of a CNT. The chiral vector can also be denoted using the chiral indices (*n*, *m*). The vectors are generally written as C = *n*a_1_ + *m*a_2_, where a_1_ and a_2_ are the basis vector of the graphene sheet. A nanotube is chiral if its index *m* is larger than zero and it is not the same value as the index *n*; its enantiomer will then have the opposite chiral index, as they are both mirror images.

The chiral indices also affect the chiral angle, where θ = tan^−1^ [√3(*n*/(2*m* + *n*))]. The chiral angle is useful in separating the CNTs into different classes based on their electrical properties: armchair (*n* = *m*, θ = 30°), chiral (0 < *m* < *n*, 0 < θ < 30°), and zigzag (*m* = 0, *n* > 0, θ = 0°). The armchair CNTs have electrical properties that are similar to metals, while the chiral and zigzag CNTs are quasi-metallic or semiconductors. The chiral and zigzag CNTS are quasi-metallic with a finite band gap if *n − m* is divisible by 3 and *n* ≠ *m*, otherwise they are moderate semiconductors in all other circumstances [33].

### 2.4. Graphene Oxide

Graphite oxide is a chemical compound containing carbon, oxygen and hydrogen in different ratios depending on its preparation method; it is derived from the oxidation of graphite crystals (which are easily obtainable and inexpensive). Graphite oxide that has been exfoliated into a single atomic layered strip is named GO. It is a yellow solid with a carbon to oxygen ratio between 2.1 and 2.9 that maintains its original graphitic structure but has an increased distance between its layers (around two times larger). As we know, the interlayer distance maintained due to the van der Waal forces between graphite is approximately 0.34 nm. However, the chemical oxidation will result in the disruption of the hexagonal carbon framework of graphite due to the intercalation of newly formed oxygen-containing functional groups between the layers of graphite which disrupts the delocalized electronic structure of the layers [34], which results in an increased interlayer spacing (around 0.7 nm) for graphite oxide.

Graphite oxide is produced when graphite is oxidized using strong acids and other oxidizing agents where the simple aromatic rings of the graphite are destroyed and oxygen containing functional groups (epoxy, carbonyl, carboxyl and hydroxyl groups) are introduced into the hexagonal carbon network [35]. Generally, hydroxyl and epoxy functional groups are interspersed on the top and bottom of the graphite sheets while the carboxylic acid and carbonyl groups are located at the edges of the graphite oxide sheets [36]. Thus, graphite oxide is a compound that maintains the layer structure of graphite but contains a range of oxygen-containing functional groups, along with the non-oxidized areas where the carbon atoms retain sp^2^ hybridization.

GO is then obtained through the exfoliation of graphite oxide. This can occur via thermal shocking or chemical reduction in a suitable medium. For the first method, rapid heating will cause the exfoliation and reduction of graphite oxide, causing the sheets to expand and yield GO [37]. The heat is assumed to cause several small molecules in graphite oxide to evolve, which leads to a rise in internal pressure, which spreads the sheets apart. In the second method, the hydrophilic nature and larger distance between the layers of graphite oxide (when compared to graphite) eases the direct exfoliation in water, such as ultrasonication.

Additionally, GO has two advantageous characteristics. First is the presence of oxygen-containing functional groups (such as hydroxyl and carbonyl groups), which results in GO being hydrophilic and is able to be dispersed in water. The presence of these functional groups allows it to form stable suspensions in various solvents such as water and organic solvents via electrostatic repulsion due to its net negative charge [38], which makes it easier to be processed for industry applications. The second benefit is that it has a low production cost, as graphite is an inexpensive raw material. Graphite oxide can also be produced using other cost-effective methods such as Brodie method and Hummers method that has a high yield.

This carbon nanomaterial is also used as a nanofiller, since the presence of oxygen containing functional groups aids its even dispersion in aqueous and organic solutions. This is beneficial to the process of creating a polymer nanocomposite as it can be dispersed within the polymer matrix to enhance the properties of the original polymer. Additionally, due to disruption of the sp^2^ bonds it can insulate electricity excellently. This is why GO is incorporated as nanofiller in PVC to further enhance the already good electrical insulation of PVC for cable and wiring insulation application.

To tie in with the subject matter of this review paper, functionalization of GO will alter its properties. Thus, the chemically modified GO could become adaptable for various applications. There are many ways in which GO can be functionalized, depending on the desired application. Nonetheless, GO is applicable in almost every application and should be considered the material of the future.

### 2.5. Fullerene

Fullerene can be described as a carbon molecule whose atoms are linked by single and double bonds which form a closed or partly closed mesh. The carbon molecules can take the shape of a hollow sphere, ellipsoid, tube, or other structures. Fullerenes are stable molecules made up of sp^2^ hybridized carbon atoms that are bent in order to produce the shape of the closed sphere or tube. This bending of the carbon atoms produces angle strain, which explains why the fullerenes behave similar to electron deficient alkenes as electrophilic addition will alleviate the angle strain by converting sp^2^ hybridized carbon atoms to sp^3^ hybridized atoms. This shift in the hybridized orbitals will result in the decrease of the bond angles from 120° to 109.5°, which produces a more stable fullerene molecule as the carbon bonds will bend less when forming the closed sphere or tube.

**Figure 2:**
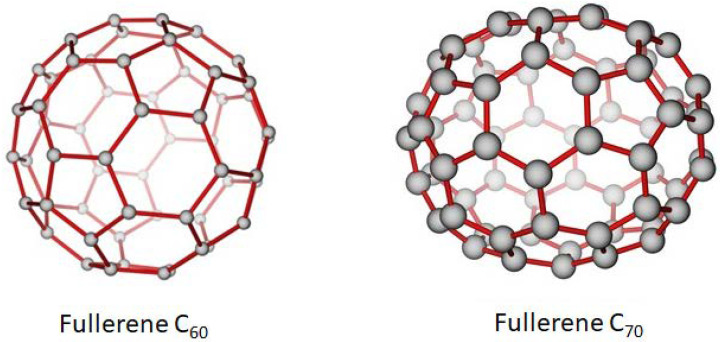
Structure of fullerene C_60_ and C_70_.

There are two major types of fullerenes: the closed fullerenes and the open-ended CNTs. Since the above section has already explored the CNTs, only the closed buckyballs shall be described here. Buckminsterfullerene (C_60_), also known as buckyballs is one of the most famous fullerenes; it also shares a similar shape with a soccer ball. Buckminsterfullerene was first discovered in 1985, which led to Harold W. Kroto, James R. Heath and Richard E. Smalley being awarded the 1996 Nobel Prize in Chemistry. C_60_ is a type of fullerene that contains 60 carbon atoms, made of 20 hexagons and 12 pentagons. It is able to withstand high temperatures and pressures [39]. The other fairly common buckyball is C_70_, whose rugby ball-like structure is made up of 25 hexagons and 12 pentagons. Both of these fullerenes have similar characteristics. The structure of C_60_ and C_70_ is shown in Figure 2.

Ever since it was discovered, fullerenes have received intense research which leads to the discovery of certain unique properties; for example, while fullerenes are generally electrical insulators, doping it with alkali metals will result in a conducting or even superconducting material, as tested by Hebard [40]. Fullerenes also display high mechanical stability, transformation into ferromagnets when doped with organic reducing agents, being chemically active and conversion into diamonds under high pressure and temperature [41].

Although fullerenes and their families have numerous unique properties, not many of these properties have been fully exploited in the industry. One of the reasons is that fullerenes such as pristine C_60_ are difficult to be processed. For instance, the solubility of most fullerenes is generally quite low. This can be seen in the case of pristine C_60_, which is insoluble in water and practically insoluble in methanol; with solubility values of 1.3 × 10^−11^ and 3.5 × 10^−5^ mg/mL, respectively [42]. This lack of solubility in water and other solvents raises some difficulty when it comes to applying fullerenes in certain industry, such as medicine. This flaw in the properties of fullerene can be rectified by functionalization. For example, attaching polar functional groups such as carboxyl and hydroxyl groups to the fullerenes would render it water soluble. Another advantage is that the reactivity of the fullerenes is increased when functional groups are attached to their surfaces.

However, functionalization of fullerenes also has its disadvantages, which brings us to another topic in this review paper: polymer nanocomposites. One of the drawbacks of functionalization is that the incorporation of functional groups tends to cause the fullerenes to agglomerate leading to large clumps of fullerenes, which may result in the poor properties that were found in the polymer nanocomposites such as decrease in tensile strength and impact strength. Nevertheless, adding fullerenes as nanofillers into a polymer can help increase the processability of this new composite (as the polymer nanocomposites can be manufactured using regular equipment in a factory) and help enhance its properties, which will promote their application in many areas in the industry [43]. This can be seen in the works of Zuev et al. [44], where the in situ polymerization of polyamide-12 combined with the addition of 0.02–0.06 wt% of pure C_60_ fullerene as the nanofiller resulted in an increase of both the Young’s Modulus and tensile strength by 30–40% when compared to pure polyamide-12. It is also worth mentioning that addition of fullerene nanofillers has the least effect in rigid polymer matrices, while it is more noticeable in plastic polymers [45].

To summarize this section, a comparison between the various properties of the above carbon nanoparticles are tabulated in Table 1.

### 2.6. Applications of Carbon Nanoparticles

As mentioned above, carbon nanoparticles possess many unique properties that have caused it to receive attention from the scientific and engineering community. This in turn has spurred the development of carbon nanoparticles so that it can be applied in a variety of industries. One of its applications is in the area of energy storage. This is due to its high electrical and thermal conductivity that renders it an ideal material to be used in energy storage systems such as capacitors or batteries. For example, CNTs have been added to the electrode catalyst mixture in order to enhance the catalytic reaction in the fuel cells and to reduce the use of precious metals. Furthermore, functionalized graphene is also part of the material used to produce fuel cells and batteries [47].

Carbon nanomaterials can also be used for bioimaging applications. For example, graphene based nanoparticles have been used in optical imaging, radionuclide-based imaging, magnetic resonance imaging (MRI), computed tomography (CT) scan and multimodal imaging, due to its versatile surface functionalization and extremely high surface area [48]. One specific example is nano-graphene oxide, which is used in fluorescence imaging due to its inherent photoluminescence.

Lastly, carbon nanoparticles can be used for environmental sensing, where they detect the presence of pollutants. This is seen through the use of CNT based sensors, where the excellent electrical conductivity, large surface area, rigidity, and chemical stability all serve to improve the original carbon electrode sensors [49]. For example, the nanotubes can help enhance the sensitivity of the sensors; this occurs when charged species of the sample are adsorbed to the surface of the nanotubes, which causes the current to fluctuate, which means a correlation can be drawn up between the changes in the current and the composition of the sample. CNTs are also able to produce electric fields at low voltages, which allows for the creation of a small-sized sensor that is fully powered by batteries.

## 3. Synthesis of Carbon Nanoparticles

### 3.1. Synthesis of Carbon Nanosheets

Carbon nanosheets are most frequently produced using the bottom-up method, which includes chemical synthesis methods such as solution phase synthesis and CVD. The other commonly employed strategy is the top-down method which includes the exfoliation of graphite such as chemical and mechanical exfoliation [50]. Due to the large number of methods used to synthesize carbon nanosheets, this section will only discuss three methods which are chemical exfoliation and mechanical exfoliation, thermal decomposition and CVD.

#### 3.1.1. Mechanical and Chemical Exfoliation

Exfoliation is a process that occurs when external forces manage to successfully overcome the van der Waal forces between the graphite layers. This is because the strength of the van der Waal force is inversely proportional to the distance between the layers. Some of the methods used in order to increase the distance between the adjacent graphite layers are through mechanical or chemical means such as oxidation. To explain the process, the chemical oxidation of graphite will result in functional groups such as hydroxyl or carbonyl groups to be attached to the layers, causing disordered graphitic stacking and increased spacing between the layers; all of which will expedite the exfoliation process [51]. In general, exfoliation is a process where a material expands up to a few hundred times along an axis.

Mechanical exfoliation of graphite is able to occur at a force of 300 nN·μm^−2^ because the van der Waals force between the layers of the graphite is around 2 eV·nm^−2^ [50]. Due to the low amount of force required, it is a relatively straightforward method to create carbon nanosheets directly from graphite. However, this method has a few disadvantages, namely: unable to be produced at a large scale, poor reproducibility, and the fact that mechanical exfoliation may cause agglomeration of graphite filler or damage to the nanofilllers. An example of this process is to place graphitic carbon nitride (g-C_3_N_4_) in water and sonicate the mixture to obtain carbon nitride nanosheets [52].

The other form of exfoliation is chemical exfoliation. The advantage of using chemical exfoliation is that it is successful in producing thinner carbon nanosheets, which is beneficial as it will lead to higher conductivity and higher aspect ratios. Additionally, the van der Waals force between the layers of these nanosheets is weaker than that of its precursors. In recent years, graphite oxide is being chosen as the starting materials to create carbon nanosheets; this is because the graphite layers are easier to be expanded and exfoliated after contact with strong acids due to oxidation. When the carbon nanosheets produced by this method are used in polymer composites, electromagnetic attenuation will be improved upon due to the good electrical conductivity of the nanosheets. This can be seen when Song et al. [53] recorded the conductivity of carbon nanosheet with thickness of 2–5 nm produced via chemical exfoliation of graphite to be 3.1 × 10^4^ Sm^−1^, compared to that of a functionalized GO sheet (2 × 10^2^ Sm^−1^) [54].

**Figure 3:**
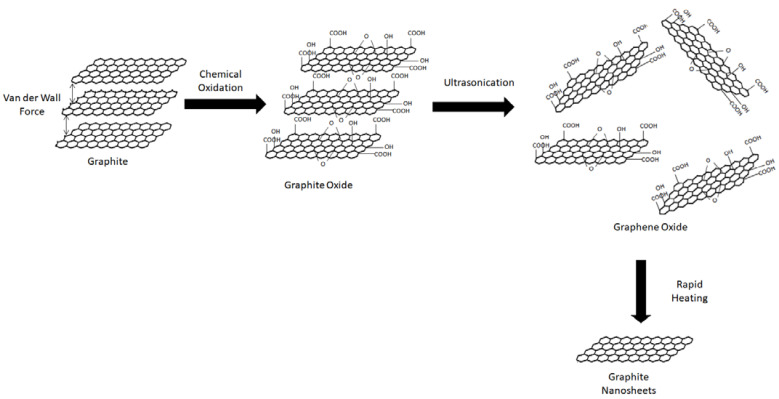
Chemical exfoliation of graphite to graphite nanosheets.

Additionally, exfoliated graphite or graphite oxide also plays a part in producing polymer nanocomposites with better properties. For example, Fukushima et al. [55] managed to synthesize nylon and polypropylene composites with enhanced thermal conductivity using the carbon nanosheets as the nanofiller. This is proven when the thermal conductivity of the polyamide-6/exfoliated graphite composite reaches its highest value of 4 Wm^−1^ K^−1^ at 20 vol% graphite loading, compared to that of the control polyamide-6 (with only 0.25 Wm^−1^ K^−1^). The chemical exfoliation is performed via oxidation of graphite by sulfuric and nitric acid, with the nitric acid performing as the oxidizer while the sulfuric acid will intercalate the graphite layer. The graphite is then exfoliated when rapid heating is applied because the layers will expand, which is caused by the vaporization of intercalated acid within the graphite. Finally, the created carbon nanosheets are then dispersed into the polymer matrix. The exfoliation process is described in Figure 3.

#### 3.1.2. Thermal Decomposition

Thermal decomposition is attained using pyrolysis. Pyrolysis is a thermochemical treatment of organic products that takes place at very high temperatures in an inert atmosphere. Through pyrolysis, carbon nanosheets could be created from organic materials such as acids, biomass and polymers. Carbon nanosheets are formed from the carbon that is produced from the thermal decomposition of the carbon precursors through the self-assembly process. To give an example of an organic compound used for thermal decomposition, citric acid will be used. For this process, citric acid was mixed with an excess of urea, at a weight ratio of 1:10, then a gel-like substance will be formed. This gel will then be heated at 350 °C for 4 h followed by annealing process at 650 °C for 10 h. The carbon formed from the thermal decomposition of nitric acid is greatly dispersed, which will prevent agglomeration of the decomposed carbon and result in an ordered structure for the carbon nanosheets [56]. The thickness of these nanosheets is less than 20 nm. Pyrolysis of folic acid can also result in carbon nanosheets. Folic acid was carbonized at 900 °C, which will result in porous carbon nanosheets that have a thickness of around 900 nm. The benefits of using thermal decomposition are that this method does not require the use of environmentally harmful surfactants or toxic chemicals, is straightforward and cost effective.

The carbon nanosheets created by pyrolysis can also be used to create polymer nanocomposites as seen in the works of Su et al. [56] with NiCl_2_·6H_2_O being added to the carbon nanosheets to create Ni/C nanocomposite with enhanced properties. This is seen when the well dispersed Ni/C nanosheets resulted in a composite with impressive reversible capacity (1051 mAh g^−1^ after 30 cycles), larger than that of regular graphite (372 mAh g^−1^) or nitrogen doped carbon nanosheets [56].

Industrial hemp phloem fibers can also be used as a starting material for the pyrolysis. Firstly, the hemp fibers are heated at a temperature around 180 °C for 24 h. The resulting substance is then heated again at a higher temperature, which results in exfoliation of the hemp fibers into the carbon nanosheets [57]. This will produce a carbon nanosheet that is partially graphitic, highly mesoporous, with a thickness of 10–30 nm and acceptable electrical conductivity (up to 226 Sm^−1^) [58]. The hemp-derived nanosheets can then be used as an electrode while an ionic liquid functions as the electrolyte in order to create a functioning supercapacitor. The created supercapacitor has outperformed the ones in the market, with energy densities reaching up to 12 W·h/kg (2–3 times higher than commercial supercapacitors). The operating temperature range is also very large, from 0 to over 90 °C. While graphene can also be used in the creation of supercapacitors, the pyrolysis method is much cheaper as graphene is expensive. Other carbon precursors besides acids and biomass that could be used in the pyrolysis method include peat moss, ethylene glycol, chitosan and urea.

#### 3.1.3. Chemical Vapor Deposition

CVD is another method that can be used to prepare carbon nanosheets. To carry out this method, a gaseous carbon precursor is treated at high temperature which results in the decomposition and subsequent aggregation of the precursor molecules. These aggregates will further grow due to the continued breakdown and deposition of the precursor molecules, which will result in the creation of carbon nanosheets. The carbon sources that can be used for CVD include methane and acetylene gas. The advantage of using CVD is that it is a relatively straightforward process. Moreover, the carbon nanosheets produced using CVD have a high degree of graphitization, good structural regularity and high purity [15].

There are different types of CVD available, such as the plasma enhanced CVD, which can be used for the formation of various carbon nanomaterials, such as CNTs and graphene films. Other types include the microwave plasma CVD, hot-filament CVD and radio frequency plasma enhanced CVD. For example, carbon nanoflakes were successfully grown on a silicon substrate via hot-filament CVD using methane gas as a carbon precursor [59]. The CVD reaction occurred at 840 °C for 2 h in a H_2_/Ar/methane precursor gas mixture, with a flow rate ratio of 15/84/1. Additionally, the chamber pressure was kept at 1000 Pa while the substrate temperature was maintained at 2400 °C.

Furthermore, a heat treatment process in the range of 1200–3000 °C of the carbon nanosheets has the added effect of removing the amorphous carbon phase and other defects, which would improve the graphitization degree of the nanosheets. The degree of graphitization increases while the interlayer spacing decreases with increasing temperature. The edges of the carbon nanosheets annealed at 2100 °C and above are composed of 2–5 layers of graphene with many zigzag functions [60]. The high temperature treatment is also shown to increase the electrical conductivity of other carbon nanomaterials, which will reduce its electrical impedance when used as an electrode [61].

### 3.2. Synthesis of Graphene Oxide

As mentioned before in Section 2.4, GO is created from graphite that undergoes oxidation which results in the graphite layers being interspersed with oxygen molecules, which is then exfoliated using various organic solvents or sonication method, which will be explored here. Since GO cannot be prepared without first creating graphite oxide, it is important to understand how to prepare it. There are generally three methods used to prepare graphite oxide, which are the Brodie method, Staudenmaier method and the Hummers’ method. Since graphite oxide is created through the oxidation of graphite, all three methods require the use of strong mineral acids (nitric acid, HNO_3_) and oxidants (potassium chlorate, KClO_3_).

#### 3.2.1. Synthesis of Graphite Oxide

The Brodie method comes from Benjamin C. Brodie, a British chemist who was the first person to create graphite oxide in 1859 through the treatment of graphite with potassium chlorate and fuming nitric acid [62]. The procedures for this treatment are as follows: firstly, graphite along with three times its weight of potassium chlorate (KClO_3_) is added into a retort. Fuming nitric acid is then added to the mixture until it becomes a fluid so that the graphite is oxidized. The oxidation process is repeated for four or five times until the black graphite becomes a light yellow solid.

After 1859, various methods have been created to improve the synthesis of graphite oxide. One of which is the Staudenmaier method, created in 1898 by L. Staudenmaier [63]. Staudenmaier improved on the Brodie method by introducing concentrated sulphuric acid into the graphite mixture in order to increase its acidity. Another change that Staudenmaier made was separating the potassium chlorate (KClO_3_) into several aliquots and then adding the portions into the mixture over the course of the oxidation reaction [64]. Due to these alterations, the graphite oxide was highly oxidized, however, the final product still retains the same composition as the one Brodie produced. The Staudenmaier method also allows the oxidation process to be carried out in one single reaction vessel, which greatly simplifies the graphite oxide synthesis process. Nevertheless, there are some drawbacks to this method; one of which is that the gradual addition of KClO_3_ typically lasted over a week, which is bad in terms of safety as KClO_3_ is a volatile substance that may cause explosions. Another disadvantage is that chlorine dioxide will be produced due to the addition of KClO_3_, which needs to be absorbed by an inert gas, as it is a health hazard. The oxygen-containing functional groups that were found within graphite oxide created using the Brodie and Staudenmaier methods were generally carbonyl, ketone and lactone groups [65].

The last method would be the conventional Hummers’ method which was developed by William S. Hummers and Richard E. Offeman in 1958, as a safer, faster and more reliable method compared to the Brodie and Staudenmaier methods [66]. Hummers’ method is performed via the addition of potassium permanganate (KMnO_4_) to a solution of graphite (which replaced KClO_3_), sodium nitrate (NaNO_3_) and concentrated sulphuric acid (H_2_SO_4_). The whole process is performed at a temperature below 45 °C and can be completed within two hours. The graphite oxide produced via this method also has a higher oxygen content compared to the Staudenmaier method, which is shown in Table 2.

#### 3.2.2. Exfoliation of Graphite Oxide

While GO and graphite oxide are created using a similar chemical process, the difference between them lies in their structure (namely their number of layers), with graphite oxide being a multilayer system of GO, while the exfoliation of graphite oxide will produce a monolayer sheet called GO. Two common methods that are commonly used to exfoliate graphite oxide are solvent-based exfoliation and thermal exfoliation. The properties of graphite oxide such as it being hydrophilic and having an increased interlayer spacing (when compared to graphite) helps facilitate the direct exfoliation in water or organic solvents, which is then followed by mechanical exfoliation (ultrasonication or stirring). This results in a colloidal suspension of GO. While ultrasonication is a much faster process than physical stirring, it may fragment the GO platelets and lead to a reduction of its size; from a few microns to a few hundred nanometers per side [65]. On the other hand, mechanical stirring yields platelets of a larger dimension but it is a slow process with a low yield.

Exfoliation of graphite oxide can also be carried out by rapidly heating. For this method, dry graphite oxide powder is put into a quartz tube and rapidly heated to very high temperatures (400 °C or more). The thermal shock will cause the small molecules such as carbon dioxide (CO_2_), carbon monoxide (CO) and water to escape and increase the internal pressure, resulting in the sheets being forced apart [23]. The GO created through thermal exfoliation was reported to have a lateral dimension similar to those exfoliated by ultrasonication (several hundred nanometers) [67]. Thermal exfoliation can also be carried out by microwave radiation, which produces microwave-expanded graphite oxide. It is also worth noting that both these methods exfoliate graphite oxide as well as reduce it [68].

This is useful as reduced GO possesses good electrical conductivity, favorable mechanical strength and large surface area, qualities which make it attractive to be used in producing conductive polymer nanocomposites [69]. For instance, Pham et al. [70] developed a poly (methyl-methacrylate)/reduced GO (PMMA/RGO) composite with a maximum conductivity of 64 Sm^−1^ with a loading of 2.7 vol% of RGO and a percolation threshold of 0.16 vol%. It is worth noting that a material with conductivity more than 1 Sm^−1^ is acceptable to be used in most electrical appliances.

### 3.3. Synthesis of Carbon Nanotubes

CNT is a material that exhibits excellent electrical conductivity, tensile strength and thermal conductivity. CNTs also possess the ability to undergo chemical modification, such as functionalization. As a result of these valuable properties, researchers have conceived different methods in order to produce CNTs out of several carbon precursors. The three most widely used synthesis method of CNTs are electric arc discharge, CVD and laser ablation [71].

#### 3.3.1. Electric Arc Discharge

Electric arc discharge is a process where electricity is used to break down a gas in order to produce plasma. This method was first used by Iijima to synthesize fullerenes and CNTs in 1991; it also has the honor of being historically, the oldest method used for the synthesis of CNTs. The reason the arc discharge method is prevalent is that the yield for this technique reaches 30% by weight and has the ability to create both SWCNTS and MWCNTs with little to no structural defects while being both simple and inexpensive. However, this method produces nanotubes that are rather short (50 micrometers and below) [72]. Eatemadi et al. [73] reported that the high temperature of the arc discharge technique (>1700 °C) is the reason for the expansion of CNTs with little structural defects.

The arc discharge chamber has two electrodes, the anode which consists of evaporated carbon precursors and metal catalysts, such as cobalt, nickel or iron, and the cathode, which is generally a pure graphite rod. The chamber is filled with an inert gas such as helium or argon. After turning the power on, the two electrodes are then bought into contact with one another to create an arc and are then kept at a distance of 1–2 mm in order to achieve a steady discharge. A constant current is maintained at 100 amps, which vaporizes the carbon and produces plasma with very high temperature [74]. Around half of the carbon vapor then cools down and condenses on the cathode, while the other half is deposited on the walls of the chamber and condenses into “chamber soot”. During the arc discharge process, the anode is constantly being consumed. After an arc application time of a few minutes, the power is turned off and the cathodic deposit along with the chamber soot is then collected.

There are two main types of arc discharge method, which are synthesis using catalyst precursors and synthesis without using catalysts. As a rule, MWCNTs can be synthesized without the use of catalysts. However, production of SWCNTs requires the use of catalyst precursors and a complex anode (for expansion in arc discharge). The complex anode is made from a mixture of graphite and metal, such as gadolinium, cobalt, silver and palladium [73]. The main advantage of the electric arc discharge method is that it has the capability to produce large amounts of nanotubes. However, the main disadvantage is that the chirality of the nanotubes created cannot be controlled. Another flaw is that purification of CNTs is required if metal catalysts are used in its synthesis.

#### 3.3.2. Laser Ablation

The laser ablation method is carried out by placing a pure graphite target in a quartz tube and vaporizing it using intense laser pulses under a flowing argon (or other inert gases) atmosphere. The carbon vapor is then moved by the flowing argon gas from the high temperature zone to the water-cooled surface of the nanotubes collector outside the furnace so that it can be condensed to CNT. The quartz tube is then placed in a furnace that can withstand a temperature up to 1200 °C. This method was created by Guo and his co-workers when they were finding a new method to create SWCNT [75]. Similar to electric arc discharge, metal elements have to be added to the graphite block as catalysts in order to create SWCNT.

This technique has a yield of approximately 70% and primarily produces SWCNTs, with a diameter that can be controlled by changing the power of the laser pulse, with higher laser power decreasing the diameter of the CNTs [76]. Guo et al. [75] also discovered that the yield of the nanotubes seemed to increase with increasing temperature, until the maximum temperature of the furnace (1200 °C). Other parameters that can influence the properties of CNTs produced are structure and chemical composition of the graphite, the properties of the laser (such as peak power, oscillation wavelength and rate of repetition), flow rate and pressure of the carrier gas, the pressure in the chamber and ambient temperature.

While this method is similar to that of arc discharge, such as using a large amount of energy to vaporize graphite and let it condense in the form of nanotubes, the necessary energy is produced by a laser. The other difference is that laser ablation is primarily used to create SWCNT with high purity (reaching up to 90% purity) and high quality, as the structure is better graphitized compared to those synthesized using the arc discharge method. The reason for the lower impurity is because the metal catalysts used tend to evaporate out of the quartz tube even after it is closed. However, the main disadvantage of using this method is that its high cost prevents it from producing CNTs on a large scale, limiting this method to only be used in a lab [72]. The exorbitant price comes from the laser and the highly purified graphite block.

#### 3.3.3. Chemical Vapor Deposition

In recent years, one of the most popular methods used to synthesize CNTs is CVD. This is because unlike the arc discharge method, which can produce large amounts of unpurified CNTs, and laser ablation which only produces a small amount of carbon deposits but with higher purity, CVD offers the best of both worlds by offering a controllable method that is capable of producing CNTs with predefined characteristics on a large scale [28]. Additionally, CVD also has higher yield (up to 90%) and purity of CNTs compared to the two previous methods. CVD also possesses the ability to create nanotubes in an assortment of forms, including powder, aligned, entangled, straight, curved or helix. It also provides better control for the growth parameters of CNTs. However, in terms of crystallinity, the CNTs synthesized by CVD are inferior to that of arc and laser-grown CNTs [77].

**Figure 4:**
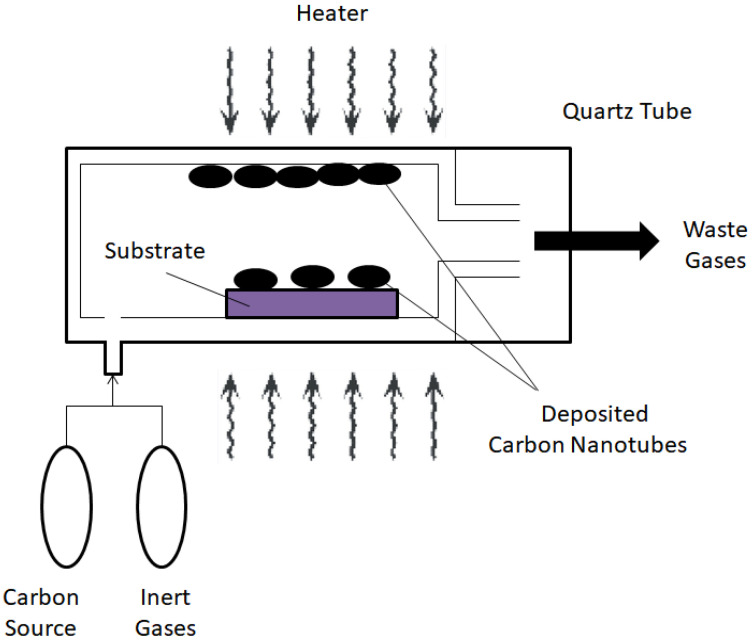
Synthesis of CNTs through CVD.

While there are many types of CVD, the one that is going to be discussed in this section is catalytic CVD as it is at present the standard technique used for the synthesis of CNTs. Catalytic CVD involves treating a gaseous carbon precursor at high temperature (600–1200 °C) by passing it through a tubular reactor where the transition metal catalyst is placed, whereby the carbon precursor will chemically broken down due to the catalyst and subsequently aggregate on the catalyst, which is then collected after the reactor is cooled down to room temperature [77]. The most frequently used carbon precursors for CNTs include methane, acetylene, benzene and xylene. In general, CVD takes place in a furnace at atmospheric pressure. There are two types of furnace modality, the horizontal configuration and vertical configuration, with the former being the more commonly used [78]. The CVD process is described in Figure 4.

The properties of the CNTs produced by CVD are influenced by certain operating parameters such as temperature and pressure of the operation, the type and concentration of carbon precursor used, the size of the metal catalyst and the reaction time [79]. For instance, Journet et al. [80] discovered that the diameter of the nanotubes can be altered by changing the active particles on the surface of the metallic catalyst. The length of the CNTs produced is also affected by the reaction time.

#### 3.3.4. Flame Synthesis Method

Synthesis of CNTs can also be done by using hydrocarbon flames. The flames provide both chemical and catalytic factors for the synthesis of CNTs and other carbon nanoparticles. During the burning, the fuel which consists of hydrocarbons such as acetylene (C_2_H_2_), methane (CH_4_) or ethylene (C_2_H_4_) will react with the oxygen in the air to create a gaseous mixture made up of CO_2_, CO, ethane (C_2_H_6_) and water vapor [81]. This post-burning gaseous mixture will serve as the carbon precursor for the CNTs. The synthesis of CNTs in flame is similar to that in CVD, as the gaseous mixture will be broken down by the catalysts which serve as the reaction site and the carbon will then be deposited on the catalysts. Transition metals such as iron and nickel can be used as the catalyst. The properties of catalysts used and carbon deposition rate will also influence the structural properties of the nanotubes produced. The flames can be scaled; this method is also used for the production of solid carbon forms such carbon black and printing ink.

Flame synthesis method could be split into two types: premixed flame synthesis and diffusion flame synthesis. The difference between the two methods is that premixed flame is produced when the fuel and oxidizer are combined before the reaction while diffusion flame occurs when the fuel and oxidant are separated prior to the combustion. The other differences are shown in Table 3. Premixed flames have an advantage over diffusion flames when it comes to CNTs synthesis. This is due to the fact that equivalence ratio can be easily manipulated by changing the mass flow rate of the fuel or the oxidant. Premixed burners also give a large surface CNT growth on a plate at the exit of the burner with wall stagnation flow [82].

In a nutshell, the advantages of using flame synthesis are that it is a rapid process and does not require high energy consumption, which makes it less costly to use. Due to these simple and economical attributes, this method offers a cost effective mass production of CNT. Additionally, to wrap up this subsection, a summary and comparison of the above synthesis methods for CNTs are tabulated in Table 4.

## 4. Functionalization of Carbon Nanoparticles

While carbon nanoparticles are known to have many unique properties, the strong van der Waals forces between the nanoparticles have restricted the manipulation of these nanoparticles and limit their use in a variety of fields. For example, most carbon nanomaterials such as CNTs are known to be insoluble in water which presents a problem as many chemical and biomedical applications require the use of aqueous solvents. To solve this issue, functionalization of carbon nanoparticles takes place. Functionalization is a process which modifies the surface of a material by introducing molecules or nanoparticles via chemical bonding, adsorption or electrostatic interaction [85]. This method can alter physical and chemical characteristics such as coarseness, hydrophilicity, reactivity and surface energy. Many organic and inorganic species can be incorporated on the nanoparticles such as proteins, polymers, metal nanoparticles and amines. The types of functionalization that will be expanded on are covalent functionalization, non-covalent functionalization and supercritical fluid (SCF) treatment.

### 4.1. Covalent Functionalization of Carbon Nanoparticles

Covalent functionalization is the process where chemical species are grafted on the surface of carbon nanoparticles through the formation of covalent bonds, which share at least a pair of electrons between the carbon nanomaterial and the incorporated chemical species. While this method will cause defect sites to be created within the conjugated structure of the functionalized carbon nanoparticles, it generally produces stable and controllable bonds.

#### 4.1.1. Covalent Functionalization of Graphene

Functionalization is generally performed on graphene to resolve some of its shortcomings. Some of these flaws include easily agglomerating with one another via π-π stacking between multiple graphene layers and van der Waal force [86]. Functionalization can also reduce the surface energy of graphene which increases its stability, for instance by grafting a polymer to graphene. Another property of graphene that can be enhanced using functionalization is opening its band gap, which allows the functionalized graphene to be used in electronic applications [87]. The band gap can be controlled by manipulating the conjugation length of the sp^2^ hybridized lattice through covalent functionalization.

Graphene is fairly chemically inert (it has lower reactivity than fullerenes and CNTs) which is due to the giant delocalized π bonding system. Thus, most covalent functionalization of graphene is done by using reactive chemicals that are able to form covalent bonds with the sp^2^ hybridized carbon atoms [88]. Some of these reactive species include free radicals, nitrenes and carbenes. For example, graphene was treated using (p-nitrophenyl)-diazonium tetrafluoroborate which resulted in aryl groups to be covalently bonded to the surface of graphene. The reaction happened due to the donation of electrons from the graphene to the aryl diazonium ion which creates an aryl radical after nitrogen gas (from the chemical reaction) is purged from the reaction [89]. The presence of covalent bond was also detected by X-ray photoelectron spectroscopy (XPS). Sharma et al. [90] reported that functionalization with diazonium salts has resulted in the reactivity of graphene edges being increased at minimum 2-fold when compared with single layer graphene. The reactivity was measured using Raman D/G ratio, with the intensity of disorder D peak for graphene being compared before and after functionalization. Diazonium functionalization has also managed to enhance the electrical properties of graphene, with the covalent bonding of the aryl group to the basal plane of graphene resulting in part of the sp^2^ bonded carbon on graphene being converted to sp^3^ ones, and the change in the conjugation length of delocalized carbon lattice. It was also reported that increased conductivity would occur only with the use of the higher concentration of diazonium salts and a longer reaction time as this would result in graphene being functionalized to a higher degree. The solubility of pure graphene was also enhanced using diazonium functionalization [91].

#### 4.1.2. Covalent Functionalization of Carbon Nanotubes

While CNTs possess many unique characteristics as mentioned in Section 2.3, they still shares some flaws that are seen in graphene, such as frequent agglomerating with one another through π-π stacking and strong van der Waal force. CNTs are also insoluble in most solvents. As such, functionalization is used to resolve some of its drawbacks. There are two types of covalent functionalization for CNTS, which are (1) direct covalent method, which takes place on the sidewall of CNT where the sp^2^ hybridized carbon atoms are changed to sp^3^ ones, and (2) indirect covalent method, where carboxyl groups and other oxygen containing functional groups located on the open ends and holes in the CNT sidewall are chemically altered. The carboxyl groups either occur naturally on the surface due to CNTs synthesis or produced during oxidation reaction by oxidizing agents, such as nitric acid, oxygen and concentrated sulfuric acid. The hydrophilic functional groups are necessary in order to increase reactivity and solubility. These functional groups will then be converted to acid chloride via esterification or amidation reaction [92].

One example of covalent modification to the surface of SWCNT is through aniline functionalization via amidation [93]. The process starts with the functionalization of SWCNT using 50 mL of oleum and nitric acid each to create carboxyl functional groups on the surface of the nanotubes. The acid functionalization serves to open the ends of the nanotubes and produce oxygen-containing functional groups. The acid mixture was then irradiated with microwave radiation, which results in SWCNT with increased solubility. The reason for this is because microwave irradiation would result in the formation of a significant number of hydrophilic groups [94]. The soluble SWCNT was then mixed with hydrochloric acid (HCl) in order to transform the carbonyl groups to carboxyl groups. The soluble SWCNT was then sonicated with oxalyl chloride before being furthered functionalized with aniline. The addition of the mineral acid resulted in the covalent bonding between the carboxyl functional groups and the aniline through acid catalytic mechanism at ambient temperature.

#### 4.1.3. Covalent Functionalization of Graphene Oxide

GO does not share similar problems with solubility in aqueous solution when compared to other carbon nanoparticles such as graphene, CNTs and fullerenes as it contains hydrophilic functional groups (carbonyl, carboxyl and hydroxyl) on its edges and basal planes [95]. However, this limits the use of non-polar organic solvents as GO has strong hydrophilicity, which causes poor dispersion and low stability in those solvents. For instance, exfoliation of GO is difficult to carry out in organic solvents as the strong hydrogen bonds between adjacent GO layers stop the organic molecules from entering the interlayer spaces, which prevents complete exfoliation of GO [96]. It also prevents the preparation of GO-polymer composites in organic solvents. One of the methods that can weaken the hydrophilicity is functionalization, where the oxygen containing functional groups can react with other chemical species, to decrease the number of hydrogen bond donors on the surface of GO.

Polymers can be attached to carbon nanoparticles using the “grafting from” approach. This method generally has three steps, which are incorporation of the necessary functional groups on the surface of the nanoparticles, followed by covalent modification of the initiator molecule from the functional groups and lastly, the grafting of polymers from carbon nanoparticles via polymerization methods [97]. One example is the incorporation of polynorbornene (PNB) onto the surface of GO sheets using surface initiated ring-opening metathesis polymerization (SI-ROMP) [98]. The many hydroxyls or other oxygen containing functional groups on GO will initially react with the norbornene acid chloride and form covalent bonds through esterification. A very effective Grubbs catalysts will then be employed onto GO, which will produce a ruthenium (Ru) catalyst functionalized GO (GO-[Ru]). This modified GO will be used during the SI-ROMP polymerization of norborene monomers to create PNB functionalized GO. The functionalized GO was found to have increased solubility in organic solvents such as chloroform and acetone, which indicates the success of covalent modification of GO.

Another example of covalent functionalization of carbon nanomaterials with polymer is the one with graphene using polysaccharides. The effect of this functionalization is the reverse of the above GO in that instead of making graphene more hydrophobic, the incorporation of hydroxypropyl cellulose increases its solubility in aqueous solutions [99].

### 4.2. Non-Covalent Functionalization of Carbon Nanoparticles

While much progress has been made for the covalent functionalization of carbon nanoparticles, there are some disadvantages to using this method. For example, covalent chemical modification of CNTs has shown a decrease in mechanical strength and electrical conductivity when compared to pristine CNTs [100]. Additionally, the structure of the CNTs would be altered due to sonication used during some functionalizations.

Non-covalent functionalization does not involves forming covalent bonds but is instead carried out via various electromagnetic or adsorption forces including van der Waal forces, hydrogen bonds, π-π stacking, and electrostatic force [92]. In other words, this functionalization uses intermolecular forces other than covalent bonding. One benefit of non-covalent functionalization is that is that it the electronic structure of the carbon nanoparticle is not disrupted and the structure is maintained in its original shape. On the other hand, some of its disadvantages include being difficult to control and characterize. The non-covalent functionalization also used intermolecular forces that are weak, which caused some applications to not be implemented. However, this weak bonding has caused this functionalization to be a reversible process, where reversible detaching is able to transpire easily.

#### 4.2.1. Non-Covalent Functionalization of Graphene

During non-covalent functionalization of graphene, the physical properties are conserved as additional defects are not created and the chemical structure of sp^2^-hybridized carbon lattice is not notably disrupted when compared to original graphene, which is useful if certain properties needed to be preserved, such as the electrical conductivity of graphene. The surface modifying species used during non-covalent functionalization include surfactants, polymers, biomacromolecules, and polymers.

Besides conserving the properties of graphene, certain characteristics can also be enhanced such as its solubility in water. Using non-covalent interactions, stable dispersion of graphene in water was achieved by Bai et al. [101] using the functionalization with sulfonated polyaniline (SPANI). The functionalization of graphene sheet took place when a mixture of graphite oxide and SPANI was chemically reduced together using hydrazine in an aqueous solution. While the reduction of graphite oxide would generally lead to the precipitation of graphite due to the swift agglomeration of the graphene sheets, the addition of SPANI managed to disperse the graphene sheets. The stable dispersion was caused by the π-π interactions between the SPANI chains and the graphene basal planes [102] and the electrostatic repulsion between the negatively charged SPANI/reduced graphene sheets. The maximum dispersion of graphene functionalized by SPANI in water is >1 mg/mL. The functionalized graphene sheets also presented enhanced electrochemical stability and activity along with high conductivity. This is shown when the electrical conductivity of the SPANI-functionalized-graphene films prepared by filtration was around 30 Sm^−1^ [101], which is similar to that of reduced graphite oxide films.

Non-covalent functionalization can also be used to solve the poor solubility of graphene in organic solvents, which is done by attaching end-functional polymers onto graphene. This is seen when Choi and his co-workers [103] prepared organo-dispersed graphene by functionalization with amine-terminated PS (PS-NH_2_). Similar to Bai et al. [101], the graphene was created when GO was reduced using hydrazine and ammonia in an aqueous solution. The PS-NH_2_ was then dissolved in organic solvents. The organic solvent that contains the PS-NH_2_ was later added to the aqueous dispersion that contains the reduced graphene and sonicated to induce the non-covalent functionalization. The stable dispersion of graphene in various organic media occurred due to the ionic interaction between the terminal amine ions (NH_3_^+^) of the polymer and the carboxylate ions (COO^−^) of the reduce graphene. The non-covalent grafting was proven using attenuated total reflection Fourier transform infrared (ATR-FTIR) when the peak at the 1600 cm^−1^ region (signifying N-H bending from an amine group) notably broadened. The broadening indicates the ionic interaction between the terminal amine and carboxylate groups [104]. Additionally, the PS-NH_2_ polymer functionalized graphene also displayed enhanced mechanical and electrical characteristics [105]. Besides polymer, functionalization using surfactants can also be used to produce graphene that is soluble in organic solutions. For example, Liang et al. [106] synthesized a chloroform-soluble graphene via non-covalent functionalization with an amphiphilic molecule with a positive charge.

#### 4.2.2. Non-Covalent Functionalization of Carbon Nanotubes

Non-covalent functionalization of CNT is also quite similar to that of graphene, with the electronic structure and graphitic structure being maintained and the use of surfactants, ionic liquid and wrapping using polymers for the functionalization. It also has an advantage in that the reaction is carried out at comparatively mild conditions.

Aromatic molecules can be used for non-covalent functionalization of CNTs. One such example is 2,3-dichloro-5,6-dicyano-1,4-benzoquinone (C_8_N_2_O_2_Cl_2_) or DDQ, which is used to enhance the electrical properties of SWCNT by changing it from a semiconducting material to a metallic one [107]. Other aromatic organic molecules that can be used include benzene and cyclohexane. The electrical properties are modified by the coupling of π electrons between the aromatic molecule and the SWCNT [108]. The interaction between DDQ and SWCNT is comparable to that of inorganic gases [109], which is similar to van der Waals attraction and can be classified as physisorption. Zhao et al. [107] also discovered that DDQ is a strong charge acceptor to SWCNT, with a charge transfer (0.212 e) considerably higher when compared to oxygen gas (0.09 e); this indicates a strong adsorption energy, which proves that the DDQ molecule can be successfully attached to the side walls of the nanotubes. The incorporation of DDQ on the surface of SWCNT is then examined using Fermi levels. Since there is an empty level close to the valence band edge, the coupling of π electrons between the SWCNT and DDQ molecule has shifted the Fermi level into the valence band. Thus, the SWCNT is transformed from a semiconductor to a p-type conductor. This finding is also corroborated by other experiments performed, such as when SWCNT is doped with DQQ, which significantly reduced its electrical resistance.

It is also imperative to understand the difference between covalent and non-covalent functionalization, as the two functionalization methods have different effects on the carbon nanoparticles (such as structural and electrical properties), which may be favorable to the characteristics of the polymer nanocomposites that incorporate these functionalized nanofillers. The difference between the two methods are tabulated in Table 5.

### 4.3. Supercritical Fluid Treatment of Carbon Nanoparticles

A SCF is defined as a fluid that exists at a temperature and pressure that is higher or equal to its critical point. Beyond this critical point, the liquid and vapor phases are indistinguishable from one another and these two phases can coexist. As a result of this and other unique characteristics, such as possessing low interfacial tension, high diffusivity and permeability, and superb wetting abilities, it has the potential to be used in the functionalization of polymer composites. For instance, SCF treatment has the ability to deposit molecular anchors onto high surface area carbon nanoparticles due to their high diffusivity and liquid-like density [110]. This is shown when Fifiled et al. [111] managed to attach pyrene-based molecular anchors on to the surface of MWCNTs using supercritical CO_2_ deposition, such as 1-bromoacetyl pyrene (BAP).

#### 4.3.1. Supercritical Carbon Dioxide Treatment

In present times, supercritical CO_2_ has been the subject of extensive research as a medium for preparation and functionalization of carbon nanoparticles. This is because it is relatively inexpensive, safe to handle, non-flammable and has zero risk of depleting the ozone layer. Additionally, using CO_2_ has the benefit of removing unreacted materials and side products from the finished product quite easily; as they can be simply removed by flushing the system with CO_2_ fluid. CO_2_ gas also has an easily attainable supercritical state, with its critical temperature, T_c_ at 304.1 K (31.1 °C), and the critical pressure, P_c_ at 7.377 MPa [112], which is advantageous as high temperature will result in impurities such as soot and encapsulated carbon clusters.

An example of this supercritical CO_2_ functionalization is when different types of metals are successfully attached on the surfaces of carbon nanoparticles due to the chemical processes in SCF solution [113]. This is shown when MWCNTs are successfully incorporated with metal particles (such as Pd) that act as functioning metal catalysts after the Pd(hfa)_2_·*x*H_2_O (hfa being hexafluoroacetylacetonate) precursor were reduced via hydrogen reduction with supercritical CO_2_ acting as a medium [114]. TEM images confirmed that Pd particles were well-dispersed and attached to the external walls of MWCNTs. Additionally, the use of supercritical CO_2_ as a medium has resulted in stronger adhesion of the metal particles on the functionalized MWCNTs. Other than the increased adhesion, the homogenous dispersion of metal particles on the surface of MWCNTs is a result of the stabilization effect due to the strong interaction between the functional groups and the incorporated metal nanoparticles and the absence of surface tension [115] of supercritical CO_2_ that causes superior wetting of the CNTs during attachment of metal nanoparticles.

Besides metal nanoparticles, metal oxide films such as cerium(III) oxide (Ce_2_O_3_) aluminum oxide (Al_2_O_3_) can also be incorporated on the surface of CNTs after the film precursors (generally metal nitrates) were decomposed in supercritical CO_2_ as seen in the works of Sun et al. [116]. However, the SCF has to be first modified with ethanol before the CNTs can be coated with a uniform layer of metal oxide. The SCF treatment proved successful when TEM images showed that the outer layer of the CNT has been coated with a thin layer of metal oxide. The electron diffraction (ED) pattern of the coating also revealed its highly crystalline nature. The presence of the supercritical CO_2_ played an important role in the coating of the CNTs [117]. This is because while the ethanol functions as the solvent for the metal nitrate precursors, it was the CO_2_ that helped tune the properties of the solvent. The ethanol-CO_2_ fluid would then attain supercritical state under the experiment’s condition, which would allow the dissolved precursors to attach themselves on the surface of the CNTs. After that, the precursors on the CNT would then break down into metal oxide clusters and then nucleate on the surface. This would result in a uniform and continuous metal oxide nanoparticle layer.

Coating of MWCNTs with polymer has also been performed by Wang et al. [118]. The polymer in question is fluorinated graft poly (methyl vinyl ether-alt-maleic anhydride) (F-g-PMVE-MA). It was chosen to be used in the experiment because it is an electrical insulator, soluble in supercritical CO_2_, and it is resistant to being dissolved in usual organic solvents. High-resolution transmission electron microscopy (HRTEM) revealed that MWCNTs are being coated with an amorphous layer of the polymer, with its internal cavity remaining untouched. The HRTEM images also proved that the polymer and MWCNTs did not produce a bulk composite. The polymer coating generally has a thickness of 2 nm. Additionally, as the pressure of the supercritical CO_2_ decreases to the cloud point, the polymer molecules with higher molecular weight will preferentially deposit themselves on the surface of the nanotubes. This characteristic during isothermal pressure decrease is used during the supercritical fractionation of polymers [119].

#### 4.3.2. Supercritical Water Treatment

Another example of a SCF that is used in treating carbon nanoparticles is supercritical water. Supercritical water exists when water has reached the temperature and pressure above the thermodynamic critical point (T_c_ = 647.1 K, P_c_ = 22.1 MPa) [120]. Supercritical water as a fluid has many unique properties. For example, the fluid has the ability to shift its density from that of vapor phase to liquid phase at ambient conditions without changing phases and has high gas-like diffusion rates along with increased collision rates [121]. Additionally, it can act differently as a solvent compared to regular water, as supercritical water possesses excellent oxidation, diffusion and solubility abilities [122]. The advantages supercritical water has over different supercritical solvents like CO_2_ or toluene is that water is easy to obtain, an inexpensive reaction media, recyclable, non-toxic and relatively easy to handle.

One of the more appealing functional groups that are chosen to be attached on the surface of carbon nanoparticles are primary amine groups, as their incorporation on the carbon substrate will render it to be more hydrophilic and reactive [123]. Additionally, primary amines are more chemically stable than secondary amines. This was carried out by Chun et al. [124] by functionalizing water soluble CNTs via supercritical water oxidation. The success of the amine-functionalization is proven when bands on FTIR spectra indicate the presence of the amine functional group. For example, the IR bands at 1649 and 1556 cm^−1^ specify N-H bending, while the band at 1212 cm^−1^ represents C-N bond stretching. Additionally, the band between 3400 and 3250 cm^−1^ is because of the -NH_2_ stretch of the amine group. The supercritical water treated CNTs were also found to have increased dispersibility in polar solvents (water and 1-propanol) due to the presence of hydrophilic amine groups on the surface. Furthermore, the partial oxidation due to supercritical water may also give rise to the presence of carboxyl, hydroxyl, ether groups, which further increased the polarity of CNT. Functionalization also plays a role in preventing CNT from agglomerating and removing metal particles that are used as catalyst during the synthesis of CNTs [125].

To tie in with the subject matter of polymer nanocomposites, functionalized carbon nanoparticles due to supercritical water treatment have been used in the synthesis of new composite materials. This is seen in the works of Wang et al. [8], where carbon nanosheets are treated with supercritical water in order to increase its surface activity and dispersion in the polymerization solution. This is followed by addition of carbon nanosheet to produce PS composites via suspension polymerization. The functionalization of the nanosheets helped improve the interface bonding between the nanosheets and PS matrix. It is also expected that the crystal structure of carbon nanosheets will not change after the supercritical water treatment.

The general procedure for supercritical water treatment is to add distilled water and the chemicals required for functionalization into a vessel type or tubular reactor. The reactor is then heated to supercritical temperatures of around 350–400 °C and the pressure is raised to 22 MPa.

## 5. Properties of Carbon Nanoparticles after Functionalization

While carbon nanomaterials may possess many unique characteristics, its full potential has been restricted due to its disadvantageous properties such as limited solubility and strong van der Waals force between particles. To resolve this issue, functionalization is performed in order to modify and enhance certain properties of the carbon nanoparticles. This process will also increase the potential of carbon nanoparticles to be used as nanofillers in polymer composites.

### 5.1. Mechanical Properties after Functionalization

Garg and Sinnott [100] have researched the effect of covalent functionalization on SWCNT via standard molecular dynamics simulations. The nanotubes were functionalized with H_2_C=C groups. This functional group was used in order to mimic long polymer chains. After functionalization, the nanotubes were compressed to determine the stiffness and stability of SWCNTs during deformation. The results of this research showed that the covalent functionalization reduced the maximum buckling force for the armchair nanotube (a nanotube where the tubule is encircled by a closed armchair pathway) by approximately 19.4%. This indicates that functionalized nanotubes are less stiff in the direction of the tubule axis, which increases its overall resistance to failure during deformation, as the movement of the incorporated functional group will dissipate energy during deformation which reduces the possibility of mechanical failure. This decrease in stiffness is due to sp^3^ hybridized carbon atoms being formed on the walls of the nanotubes as the result of functional groups being attached there. The experiment further showed that the buckling force experienced per 100 atoms declined when the radius of the SWCNT increases, which is consistent with prior studies [126]. This decrease in buckling force also explains the stable functional state in functionalized SWCNT with a radius larger than 0.665 nm. All in all, functionalization managed to reduce the stiffness of all the functionalized SWCNTs tested by an average of 15%.

However, not all effects of functionalization are beneficial. For example, attaching carbon or hydrogen bonds via covalent bonds on the surface of CNTs may weaken its mechanical properties [127]. This is because the formation of a covalent bond between the hydrogen atoms and the carbon atoms of the CNT will result in the distortion of the atomic structure. This distortion will greatly decrease the elastic modulus, mechanical strength and fracture strain of the nanotubes. The reason for that is the hydrogen atoms have increased the length of the C-C bonds within the CNT, with the bond length increasing with the number of hydrogen atoms covalently bonded to the nanotube. Zhang et al. [128] have shown that when the strain on CNT surpasses 11%, the length of the C-C bond with two or more hydrogens atoms attached to it will significantly increase. This induces localized deformation within that C-C bond, which leads to the bond breaking apart. This explains why increased functionalization will result in decreased mechanical strength as the C-C bonds within the nanotubes will fracture more easily. The Young’s Modulus for the CNT also decreases with the increase in functionalization, which is consistent with previous research [129].

The same effect can be seen in the carbonization and hydrogenization of graphene, as the covalent bond between the carbon or hydrogen atoms and the carbon atoms of CNT will cause the graphene plane to be displaced, resulting in the distortion of the local tetragonal structure [128]. In short, the advantageous and disadvantageous effects of functionalization on the mechanical properties of carbon nanoparticles depend on the type of chemical species used during functionalization, the type of bonding between it and the carbon nanoparticle, and the hybridization of the carbon atoms in the carbon nanomaterial.

**Figure 5:**
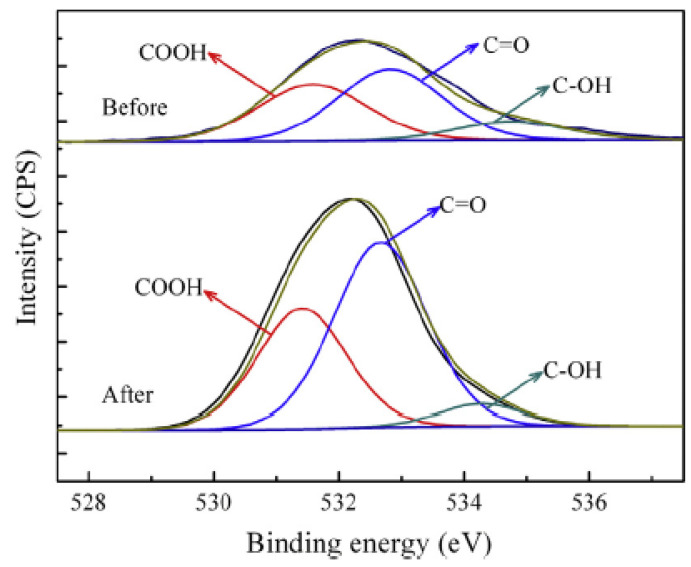
X-ray photoelectron spectroscopy (XPS) spectra of graphite nanosheet before and after supercritical water. Data obtained from [8].

Besides the effect of functionalization on the carbon nanoparticle itself, it also has the ability to improve the mechanical properties of polymer nanocomposites that used functionalized carbon nanoparticles as fillers. For instance, the beneficial effect of supercritical water treatment on carbon nanosheets can be seen in the research of Wang et al. [8] with graphite nanosheet/PS composite. After the carbon nanosheets were immersed in supercritical water, it was discovered that the number of functional groups containing oxygen has increased on the edge and surface of the carbon nanosheets, such as carbonyl, carboxyl and hydroxyl groups, due to the increase in the area under the XPS graph, which is shown in Figure 5. This is because of the moderate ability of supercritical water to oxidize carbonyl functional groups and the lone carbon atoms on the carbon nanosheets due to the increase in oxygen content within the fluid [23]. Due to this increase in the polar functional groups on the surface of the nanosheets, the ability of the carbon nanosheets to disperse in the polymerization solution has also increased as the solvent can form ion-dipole bonds with these functional groups. In spite of the functionalization, X-ray diffraction (XRD) analysis performed on the functionalized carbon nanosheets had shown that the X-ray diffraction pattern did not differ much from the untreated nanosheets as they have the same peak shape [8]. This analysis indicated that supercritical water treatment did not change the primary crystal structure of carbon nanosheets, which was later beneficial to the mechanical properties of this composite.

Due to the supercritical water treatment of the nanosheets, the mechanical properties of the carbon nanosheets/PS composites such as tensile strength and impact strength have been significantly enhanced, at 27.23 MPa and 6.12 J/m respectively [8]. This is a marked improvement over the original tensile strength and impact strength of PS, which are 12.88 MPa and 3.89 J/m, respectively. This improvement is due to the chemical bonding between the carbon nanosheets and PS, which helped transfer stress across the polymer composite interface more efficiently when the composite is stretched. Other research has shown that nano-sized fillers such as nanosheets can lessen inter-filler attraction due to their homogenous dispersion within the polymer which maximizes their potential in the polymer composites [130].

A similar effect can also be seen in the case of supercritical water treatment of carbon fibers. The mechanical properties of the treated carbon fibers have also been improved which is similar to that of the functionalized carbon nanosheets in that the interfacial shear strength (IFFS) of the carbon fiber/resin polymer composite is shown to be higher than that of the untreated carbon fiber/resin composite; which was 58.2 MPa, as opposed to 52.8 MPa. The increase in IFSS of carbon fiber/epoxy resin composite was attributed to the increase in the surface functional groups containing oxygen and roughness of the carbon fibers [131], as these functional groups will have a beneficial effect on the adhesive strength between the filler (carbon fibers) and the polymer. However, it is also worth noting that excessive treatment of supercritical water resulted in the deterioration of the carbon fibers due to excessive oxidation [120].

In summary, it was determined from the above research that functionalization of carbon nanoparticles generally has a positive effect on the mechanical properties of the material itself as well as on polymer nanocomposites after being added as nanofillers, as seen in Table 6. Additionally, the type of functionalization used also has an effect on the mechanical properties of the carbon nanoparticles; as seen in the case of Garg and Sinnott [100], where the formation of sp^3^ hybridized carbon atoms due to covalent functionalization resulted in a reduction of on average 15% to the mechanical strength of SWCNT.

### 5.2. Electrical Properties after Functionalization

Acid functionalization is one of the methods used to improve the electrical conductivity of CNTs. Functionalization takes place after MWCNTs are synthesized and it is performed using nitric acid. Singleton et al. [132] reported that MWCNTs functionalized with nitric acid have an average conductivity of 726.99 × 10^2^ Sm^−1^, which is more than twice than its untreated counterpart (236.37 × 10^2^ Sm^−1^). The electrical conductivity was measured using a four-point probe. The reason for the significant increase is due to the oxidation by the acid, which results in the formation of oxygen containing functional groups (carboxyl and carbonyl groups) on the surface of the MWCNTs, which is known to enhance electron mobility [133]. Another cause is that acid treatment in that study resulted in increased aggregation of the nanotubes, which is shown to cause increased electrical conductivity for CNT yarns [134]. Other reasons include the acid removing traces of metal catalysts used in the production method such as CVD, as the metal impurities will degrade the nanotubes’ overall properties.

Additionally, the acid functionalization increased the electromagnetic interference (EMI) shielding effectiveness of the MWCNTs, from 50.15 dB of the original MWCNT to an average value of 63.63 dB [132]. This is because of the increased thickness caused by the increased agglomeration of the CNTs. Furthermore, the EMI shielding effectiveness of both functionalized and un-functionalized MWCNTs were shown to increase with increasing thickness. This is a reasonable result as the increased thickness of the material will assist in the reflection or absorption of the EMI.

Other than acid treatment, functionalization with interhalogen compounds will also enhance its electrical properties. The halogen doping of SWCNT films with iodine monochloride (ICl) and iodine monobromide (IBr) was reported to decrease its electrical resistance by 67% and 42%, respectively [135]. This is because the addition of ICl and IBr in dichloromethane (DCM) solvent resulted in the individual nanotube and their bundles being bought closer together, which leads to capillary-induced densification. The densification reduces the sample dimension which enhances the electron transport. This is proven by the lack of high contrast regions in the SEM micrographs, as a high contrast suggests the charging of the material, and consequently poor electron propagation. The electrical resistance was measured using a four point probe [136]. Furthermore, the physisorption of the interhalogen species to the CNT film did not disrupt the network of sp^2^ hybridized carbon atoms which is advantageous as it is responsible for the conductivity of CNT.

Additionally, the electrical conductivity of polymer nanocomposites can also be increased by incorporating functionalized carbon nanostructures as filler. Wang et al. [8] reported that the conductivity of treated carbon nanosheet/PS composite increased incrementally as the average size of the nanosheets used was reduced. The carbon nanosheets were functionalized using supercritical water treatment. The highest conductivity recorded in that test was 25 Sm^−1^, while the lowest conductivity of the composite was 3.5 Sm^−1^, which was still much higher than that of untreated carbon nanosheets/PS composite (10^−7^ Sm^−1^) [8]. This improvement of conductivity is due to the carbon nanosheets possessing high aspect ratio, which is advantageous in forming a continuous conducting network as high aspect ratio particles have a high chance of interacting with one other, resulting in a material with higher conductivity [137]. The fact that the carbon nanosheets retained its crystal structure when it is homogenously dispersed within the polymer matrix also helps, as carbon nanosheet originally can conduct electricity.

In summation, it can be seen that the functionalization of carbon nanomaterials generally enhances the electrical properties of both the material itself and the polymer composite that has functionalized carbon nanoparticles added as a nanofiller. This effect can be attributed to the incorporation of certain functional groups (oxygen containing groups) and bonding of these functional groups with the polymer matrix, both of which can enhance electron mobility. Furthermore, the type of functionalization also needs to be taken into account when improving electrical properties. For example, non-covalent functionalization, which tends to be less disruptive of the electronic structure of the carbon nanoparticle is better overall for conductivity. The effect of functionalization on electrical properties of carbon nanomaterials are tabulated in Table 7.

### 5.3. Toxicity of Carbon Nanoparticles after Functionalization

While carbon nanoparticles possess unique and useful properties, their widespread usage in the industrial sector has been restricted as its toxicity and immunological effects on the human body have been recognized. For example, inhaling fine CNTs may be harmful to the pulmonary system as the CNTs may result in respiratory tract irritation and lung fibrosis [138]. However, the effects of functionalization on the toxicity of carbon nanomaterials have yet to be universally agreed upon.

Functionalization occurs when functional groups or other nanoparticles attached themselves to the carbon nanoparticles such as hydroxyl, carbonyl, carboxyl and esters groups. Most of these oxygen-containing groups are created to increase the reactivity of the substrate, as the functional groups can then react with the desired particles which will then be incorporated to the carbon nanomaterial. Thus, one way to measure the effects of functionalized carbon nanoparticles on human health is to determine its oxidation potential, which is done using dithiothreitol (DTT) assay test [139] as the presence of reactive oxygen species (ROS) is one of the factors used to determine the toxicity of particles [140]. The DTT test works by measuring the decay rate of DTT into its disulphide form when it reacts with the ROS in the functionalized carbon nanomaterial. Thus, the higher the decay rate of DTT, the higher the concentration of ROS within the sample. However, the DTT loss rate of functionalized carbon nanoparticles (SWCNT-OH, SWCNT-COOH) and its original counterparts (graphene and SWCNT) was similar to that of soot (methane soot, propane soot and diesel soot), with the one exception of GO (with 161 pmol min^−1^ µg^−1^) which was exceptionally high [141]; the results can be seen in Table 8. This indicates that GO has a higher oxidation potential than soot or other carbon nanoparticles in that experiment. Oxidative stress caused by ROS is one of the reasons for cellular toxicity [142]. This means that exposure to GO is more dangerous when compared to other functionalized carbon nanomaterials. Additionally, the difference in DTT decay rate between functionalized carbon nanoparticles suggests that the composition or structure of chemical species have an effect on their toxicities.

Another method that is used to assess the toxicity of functionalized carbon nanoparticles is by measuring the cytotoxicity to murine macrophage cells, such as murine J774 cells. The J774 cells were exposed to functionalized carbon nanoparticles while several different assays measuring the effects on several different mechanisms of cellular metabolic perturbations were performed at the same time, such as adenosine triphosphate (ATP) assay and lactate dehydrogenase (LDH) assay [142]. Liu et al. [141] discovered that the relative ATP level of J774 cells decreases with an increase in the concentration of oxidized carbon nanoparticles (SWCNT-COOH, SWCNT-OH). However, it is worth mentioning that these functionalized carbon nanomaterials have less of an effect on ATP reduction than its original counterpart, with the SWCNT having an ATP level of 0.59 when compared to SWCNT-OH and SWCNT-COOH with ATP levels of 0.93 and 0.88, respectively; the results are tabulated in Table 9. The blank sample has an ATP level of 1.01. The reduced level of ATP indicates that functionalized carbon nanoparticles are toxic, as the J774 cells are unable to metabolize in its presence.

For the LDH assay, the presence of oxidized carbon nanoparticles resulted in LDH being released at high levels. The large amount of LDH released signifies that the J774 cells may be damaged as the integrity of its membrane is compromised [143]. In summation, the ATP and LDH assay tests showed that the functionalized carbon nanoparticles have a lower toxicity regarding metabolic activity, while having a stronger toxicity in regard to membrane integrity for the J774 macrophage.

## 6. Polymer Nanocomposites with Functionalized Carbon Nanoparticles as Nanofillers

**Figure 6:**
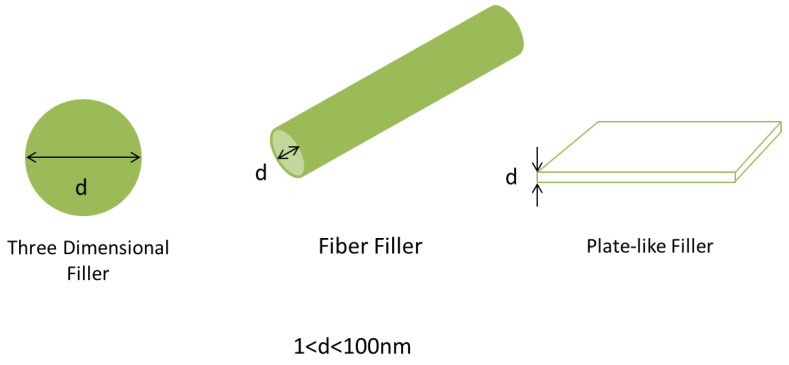
Different types of nanofillers.

Polymer nanocomposites are defined as polymers that contain nanofillers or nano-sized particles within the polymer matrix. In recent decades, polymer nanocomposites have been extensively studied as part of a novel generation of composite materials that can be used in numerous fields and applications. Based on how the polymer nanocomposite is going to be employed, appropriate nanofillers need to be chosen. For instance, carbon fibers (which are brittle yet possess high modulus) are added to polymers with low modulus of elasticity in order to create a nanocomposite that is stiff yet has low weight. While a high degree of optimization of composite characteristics has been achieved using micro-sized fillers, a new set of possibilities has been opened up in order to surpass the limitations of regular polymer composites by adding nanofillers (sheets, fibers and spheres with at least one dimension being less than 100 nm, as shown in Figure 6).

### 6.1. Polymer Nanocomposites with Functionalized Carbon Nanotubes as Nanofillers

As mentioned in Section 2.3, CNTs possess excellent mechanical, electrical and thermal properties; it is hoped that by adding them as fillers to polymer, a unique lightweight polymer nanocomposite with some of the characteristics of CNT be produced. The reason functionalization is involved in the synthesis of these nanocomposites is because of the nature of CNTs. Some of the problems include the insolubility of CNTs in most aqueous solutions, the inability to exfoliate CNT and the difficulty in improving interface bonding between the inorganic CNT and polymer matrix. Thus, functionalization (covalent or non-covalent) may provide a solution to these issues by attaching the suitable chemical species to the surface of the CNTs.

One example is the preparation of PS nanocomposites by Mitchell et al. [144] using SWCNT functionalized with 4-(10-hydroxy)decyl benzoate as nanofiller. The polymer composite was prepared via solution mixing the functionalized SWCNTs and the PS in a toluene solution. It was revealed that the functionalization of SWCNT enhanced its dispersion and chemical bonding with the PS matrix. However, it should be noted that the bond between the PS and the functional group on SWNCT are relatively weak, and it is suggested that careful manipulation of the reaction between the polymer and functionalized SWCNT will result in even greater dispersion. Additionally, the percolation threshold of the composite was observed to be as low as 1.5 wt% CNT loading. The percolation threshold is the point where the increase in filler resulted in the SWCNT particles being close enough to be in contact with one another and bring about a connected filler network [145]. Thus, once the percolation threshold is reached or exceeded, the conductivity of the PS composite will shift from an insulator to that of a semiconductor, as the continuous network would ease the flow of electrons through the nanocomposite. This increase in conductivity would be useful in applications that require conductive materials.

Besides increasing dispersion, using functionalized CNTs as nanofillers in polymer composites has the ability to improve mechanical properties. This is seen in the study of Davis et al. [146], where amine functionalized SWCNTs are used to reinforce the carbon fiber reinforced epoxy composite. The tensile test had shown that the average ultimate tensile strength and stiffness of the epoxy composite with 0.5 wt% amine-SWCNT is 747 MPa and 74 GPa, respectively, as compared to the original epoxy composite which is 681 MPa and 62 GPa. As such, the results have proven that using functionalized SWCNTs has improved the strength and toughness of the original composite. The reason behind the enhanced tensile strength of the polymer composite is suggested to be strong covalent bonding between the functionalized nanotubes and the reactive sites of the carbon fiber/fabric matrix interface. This will reinforce and consequently strengthen the fiber/fabric matrix interfaces and the nearby epoxy rich regions [147]. On the other hand, the increased stiffness is the result of strong covalent bonding between the functionalized SWCNTs and the epoxy polymer chains.

Furthermore, the reinforced epoxy composite managed to increase its durability by increasing its tension–tension cyclic fatigue life. This is because the fracture surface of the 0.2 and 0.5 wt% amine-SWCNT reinforced epoxy composite is of a ductile-type fracture and the presence of strong fiber-matrix bonding. This is proven when the optical microscopy of the materials revealed that the original epoxy composites have a brittle matrix failure while the amine-SWCNT reinforced epoxy composite possess a ductile and strengthened matrix failure. As a result, incorporating the functionalized CNTs at the surface interfaces of the fiber/fabric matrix of the epoxy composite has resulted in higher tensile strength, stiffness and durability, which will be of great use in the aerospace manufacturing industry [148].

### 6.2. Polymer Nanocomposites with Functionalized Graphene as Nanofillers

The functionalization of graphene is necessary in producing high performance polymer nanocomposites as it improves the solubility, surface activity and the dispersion of the graphene in the polymer matrix. This improvement to graphene is the reason why functionalized graphene (FG) is one of the next-generation nanofillers to be used in creating polymer nanocomposites.

One way to fabricate an FG/polymer composite is to homogeneously disperse the FG in the polymer matrix using a solvent-assisted method. For example, Xu et al. [149] managed to produce a FG/poly(vinyl alcohol) (PVA) composite film that is both mechanically strong and ductile by dissolving PVA and FG into deionized water. The FG contains various oxygen-containing functional groups on its basal planes and edges. These functional groups are responsible for the strong interfacial interactions between the nanofillers and PVA matrix. Sonication is performed to uniformly disperse the FG within the matrix. This is shown during the tensile test of the 3 wt% FG/PVA composite, which yielded a tensile strength of 110 MPa and Young’s Modulus of 4.8 GPa, which is an increase of 70% and 128%, respectively, when compared to pure PVA. Although, it is worth noting that adding too much nanofiller may cause the graphene to aggregate and result in brittleness of the material. Other studies have also shown that the mechanical properties of the composite improve with an increase in volume fraction of the nanofillers up to a certain point [150]. The composite film also shows a large elongation at break (36 ± 4%), which makes it rather ductile. The thickness of the film can also be controlled via vacuum filtration. Other polymers that have been incorporated with FG using this solvent-assisted technique include PMMA, polycaprolactone and PS [151].

FG/polymer nanocomposites can also be synthesized via in situ polymerization method. Using this technique, the monomers are polymerized in the presence of the FG. One example of this is the thermosetting of epoxy with FG as nanofiller to create FG/epoxy nanocomposite; the FG used was partially oxidized graphene, created by rapid thermal expansion of oxidized graphite oxide. Other than enhanced tensile strength and Young’s Modulus, this epoxy composite also displayed an increase in fracture toughness and fracture energy. This is shown when the epoxy composite with 0.125 wt% loading of FG managed to increase the fracture toughness and fracture energy by approximately 65% and 115% when compared to pure epoxy [152]. This increase is important as it helps increase the composite’s resistance to fracture and will prevent its failure. Since epoxy is widely used in building and construction, it is expected that this result would give rise to many applications for such epoxy nanocomposites. The reason behind this increase in fracture resistance is due to the excellent ability of graphene to deflect cracks which is related to its planar (2D) structure. The wrinkled texture of the FG is also responsible for the improved mechanical interlocking, load transfer and bonding with the polymer matrix. This wrinkled topology is due to distortions caused by oxygen-containing functional groups [153]. Another example is the synthesis of FG/PS nanocomposites using in situ emulsion polymerization. The polymerization took place when styrene monomer was added with FG, where FG was prepared by reduction of graphite oxide via hydrazine hydrate and later exfoliated in water [154]. Additionally, it is important to remember that the polymers selected for the solvent-assisted method and in situ polymerization method have to be soluble in the solvents used.

However, solvent-assisted and in situ polymerization technique is impractical for many polymers, which is why melt processing of polymer composites is the popular and preferred method in the industry. Nevertheless, the high temperature used during treatment in order to disperse the nanofillers into the polymer matrix may eliminate a majority of the oxygen-containing functional groups on the FG. Thus, it is ambiguous if these partially destroyed FG are able to form strong interfacial interactions with the polymer matrix and whether their addition would yield significant mechanical improvements. One example of a polymer composite produced using melt compounding is an FG/polycarbonate (PC) composite [155]. In this case, FG was created when graphite oxide went through pyrolysis, which resulted in it being oxidized and thermally exfoliated, resulting in the formation of FG sheets. While the electrical conductivity of the PC has been increased due to the FG forming a conductive network within the polymer matrix and its high aspect ratio, the enhancement to the mechanical properties was not substantial. While FG sheets have a higher aspect ratio than graphite, the increase to the tensile modulus by FG was similar to that by graphite nanofillers. It is suggested that high temperature during thermal exfoliation has caused structural defects in FG sheets such as wrinkling of the sheet layers and atomistic vacancies [156], which negatively affects the interaction between the FG-PC interfaces. Wakabayashi et al. [157] managed to synthesize an FG/polypropylene (PP) nanocomposite through a novel melt processing technique. The composite was produced using solid-state shear pulverization process, where both PP pellets and unmodified graphite were crushed together using a modified double screw extruder into a powder state. The high shear/compression condition resulted in the exfoliation of graphite particles into high aspect ratio nanoplatelets with around 10 nm thickness. This process also managed to uniformly disperse the graphite nanoplatelets into the PP matrix. Other nanocomposites that can be synthesized using melt blending method include FG/poly(ethylene-2,6-naphthalate) [158]. In conclusion, the melt processing method of polymer nanocomposites needs to be further developed as it is a relatively straightforward and cost effective method; only then will more polymer composites products be mass produced and commercialized.

### 6.3. Polymer Nanocomposites with Functionalized Fullerene as Nanofillers

A budding field of research is the development of Nafion composites with fullerenes and their derivatives as nanofillers. These polymer composite membranes can be used as fuel cells or humidity sensing membranes. Postnov et al. [159] showed that the Nafion composite films that used functionalized fullerenes (C_60_(OH)_22–24_ and C_70_(OH)_12_ at 1 wt% loading) as nanofillers saw an increase in proton conductivity of 10–12 times when compared to bare Nafion at 32% relative humidity (RH). Additionally, the incorporation of C_60_[C(COOH)_2_]_3_ at 1.7 wt% loading in Nafion, resulted in the highest proton conductivity at 32% RH, which is 30 times higher than bare Nafion. It is suggested that the increase in proton conductivity is due to the hydrophilic nature of the functionalized fullerene which assists in the retention of additional water in the Nafion matrix, which helps in proton migration. Furthermore, Wang et al. [160] produced a Nafion composite membrane by adding tricyanohydrofullerene, HC_60_(CN)_3_ (which is created by attaching cyano groups to the fullerene) as nanofillers into Nafion. The HC_60_(CN)_3_-Nafion composite with 1 wt% HC_60_(CN)_3_ showed a proton conductivity of 0.34 Sm^−1^ at 25% RH, as compared to recast Nafion which has 0.16 Sm^−1^. The reason for the high conductivity is due to the high acidity of HC_60_(CN)_3_, which has been increased by the attachment of cyano groups to the fullerene (an electron-withdrawing group). The increased acidity leads to the increase of proton concentration in the composite. Additionally, the larger wet and dry water uptake of the HC_60_(CN)_3_-Nafion composite is believed to be a factor in increasing proton conductivity, as it would supply more proton carriers to the Nafion composite.

Other polymer nanocomposites with functionalized fullerenes include branched sulfonated poly(arylene ether ketone sulfone) (BSPAEKS) with sulfonated fullerene as nanofillers [161]. It is reported that this sulfonated fullerene-BSPAEKS membrane with 0.75 wt% loading of sulfonated fullerene showed the highest proton conductivity (25.6–33.2 Sm^−1^) at 80 °C. The fullerene composite also enhanced water intake and mechanical strength. The incorporation of the sulfonated fullerene also had the effect of improving the methanol barrier property of the composite membrane, thus increasing its implementation in direct methanol fuel cells. The two major reasons for an increase barrier effect on methanol are as follows: the large size of the sulfonated fullerene within the polymer matrix and the hydrogen bonds between the functionalized fullerene and BSPAEKS matrix which compacts the composite membrane structure.

As a summary of Section 6, as well as to reiterate that the addition of functionalized carbon nanoparticles as nanofillers to the polymer matrix has a positive effect to the overall properties polymer nanocomposite, Table 10 shows the properties of the nanocomposite before and after addition of functionalized carbon nanoparticles.

## 7. Preparation Methods of Polymer Nanocomposites

During the last few decades, various methods for creating polymer/graphite nanocomposites have been developed. The selection of these methods is an important issue as it affects the dispersion of the nanofillers within the polymer matrix. A higher dispersion within the matrix will result in better reinforcing effects as the nanofillers will not agglomerate with one another. Some of the main techniques include in situ polymerization, solution mixing and melt blending.

### 7.1. In Situ Polymerization

**Figure 7:**
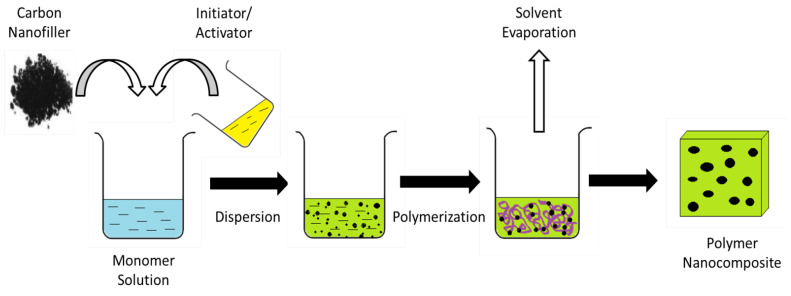
Process of in situ polymerization.

Firstly, in situ polymerization is a production method that takes place inside the polymerization solution itself. To perform this process, the fillers are first mixed with a monomer in a liquid state. Once the mixture has become homogenous, an initiator or activator is added to it; the solution is then subjected to the appropriate heat or pressure to complete the polymerization process. This polymerization process starts off with an initiation step, followed by propagation and ultimately results in various polymerization steps that lead to the fabrication of the polymer/nanofiller composite [73]. Figure 7 represents the in situ polymerization with carbon nanofillers. An example of this process is shown by Chen et al. [162], where the preparation of PS/expanded graphite nanocomposite is performed by adding the graphite filler and styrene monomer along with benzoyl peroxide as an activator in a metal vessel. The vessel was then heated to a temperature of 150 °C for 30 min and then brought down to 25 °C. Wu et al. [163] also prepared a conducting PPy/MWCNT composite by adding the pyrrole monomer to a mixed solution of MWCNT and surfactant cetyltrimethylammonium bromide (CTAB). The in situ polymerization of pyyrole on the surface of the nanotubes then occurred after adding ferric chloride as an oxidant to the previous solution.

The advantage of using this technique is that it is an effective way to disperse the graphite filers homogenously within the polymeric phase as nanofillers are present during the polymerization of the monomer or oligomer. This efficient dispersion will also result in stronger interactions between the carbon based fillers and the polymer matrix, which results in higher mechanical strength and decreased percolation value in the composite [164]. This technique is also useful for the synthesis of composites with polymers that cannot be prepared using solution mixing or melt mixing, such as polymers that are insoluble and thermally unstable. However, one of the drawbacks to this method is that polymerization occurs very quickly which limits the time window necessary to perform this process. Additionally, it is also difficult to acquire materials that are suited to this process and costly equipment is needed.

### 7.2. Solution Mixing

Another method is solution compounding or solution mixing. This technique is carried out by dissolving the polymer using a solvent with the nanofiller being added to the resulting solution. However, when faced with a carbon nanoparticle that has low solubility such as pristine CNT, mechanical stirring or ultrasonication will take place in order to achieve satisfactory dispersion of the fillers in the polymer matrix. The high power ultrasonic sonication is helpful in the dispersion, emulsifying and crushing of the nanofillers [165]. After that, the solvent is removed and the resulting polymer composite is then molded into the desired shape. An example is shown by Zheng and Wong [166] who produced PMMA/expanded graphite nanocomposite by dissolving the PMMA polymer using chloroform and adding the graphite nanofiller into the polymer matrix using an ultrasonic bath. The chloroform is then evaporated at 60 °C and the remaining composite is molded into films using a hot press. Additionally, Sahoo et al. [167] managed to uniformly disperse the functionalized MWCNT into the polyurethane (PU) matrix to prepare PU/MWNTs composites. In that work, carboxyl-functionalized MWCNT was first dispersed in dimethylformamide (DMF) solvent using sonication. Then, PU was added to the previous solution and stirred. After stirring, the solution then undergoes sonication again. The final solution is then poured onto a Petri dish and dried in an oven to produce the PU/MWCNT composite film.

The benefit of using this method is that the resulting nanocomposites possess low percolation values, which means that lower amounts of fillers are necessary to shift polymer composite from an insulator to a semiconductor. The disadvantage of this procedure is that it requires large quantities of solvent to dissolve the polymer; this has deterred the industry sector from using this method on a large scale as it may lead to environmental damage due to spilling and requires relatively large cost [164]. Furthermore, high energy sonication over a long period of time is necessary.

### 7.3. Melt Blending

The last method is melt blending. During this process, a Haake mixer, twin screw extruder and two roll mills are generally used for the blending or compounding of the polymer and nanofiller to form the composite. For example, Uhl et al. [168] had prepared a polyamide-6/expanded graphite nanocomposite by blending the polyamide and graphite filler at 250 °C using a Brabender Plasticorder mixer for a period of 10 min. Furthermore, polyamide-6 composite with functionalized MWCNT can also be prepared using melt mixing. The polyamide-6/MWCNT composite with 1 wt% carboxylate MWCNTs was prepared by blending the MWCNT and the polyamide-6 pellets at 250 °C for 10 min in a Brabender twin-screw mixer. The SEM micrographs also revealed that a homogenous dispersion of the nanofiller was achieved throughout the polyamide-6 matrix [169].

The advantage of using melt blending is that it is a straightforward process that is relatively cheap and environmentally friendly as zero solvents are required for the fabrication of the polymer nanocomposite; this makes it a favored manufacturing technique by the industry [164]. Melt blending is also commonly used for thermoplastic polymers. Additionally, composites formed using this method generally have balanced electrical and mechanical properties. However, there are some disadvantages from using the melt blending method. For instance, agglomeration of graphite filler was found in the composites after blending, which may lead to poor mechanical properties in the composite. Mechanical blending might also cause damage to the graphite nanofillers which will prevent the efficient construction of networks within the polymer composites [170]. Additionally, the melt viscosity when mixing the polymer and filler together is higher than the viscosity of the polymer and filler solution in solution compounding. This is undesirable as high viscosity will hinder the polymer chain from intercalating easily into the minor pores of the nanofillers, which will result in poor dispersion of the graphite filler in the polymer matrix [166].

To conclude this section and summarize the contents of this section, the various polymer nanocomposites synthesized by the three methods along with their benefits and drawbacks are recorded in Table 11.

## 8. Use of Polymer Nanocomposites with Carbon Nanoparticles as Nanofillers in Industry

**Figure 8:**
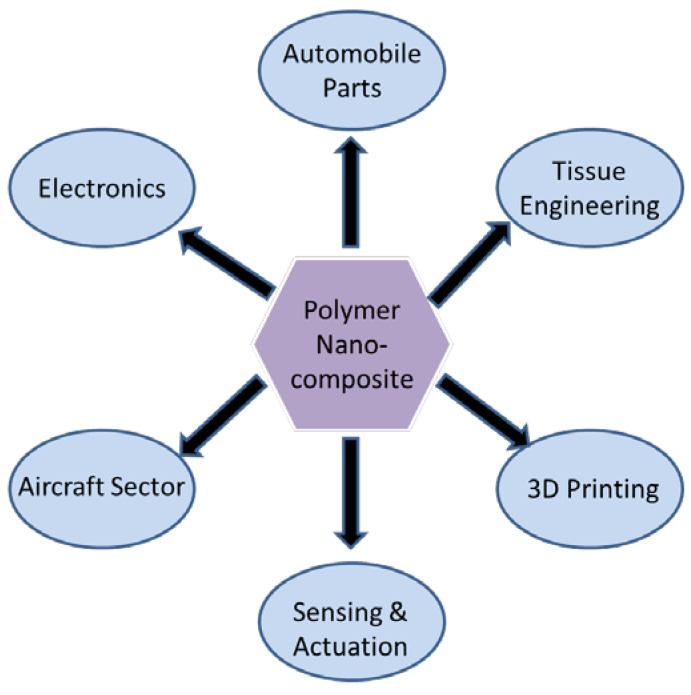
Application of polymer nanocomposites.

As established in the previous section, most carbon nanomaterials possess outstanding mechanical, electrical and thermal properties, in addition to their nanometer size and high aspect ratio. Thus, they are used as fillers in many polymers in order to enhance certain characteristics for specific applications. The applications of these polymer nanocomposites in various areas are shown in Figure 8.

In recent years, polymer nanocomposites have received attention from the automotive industry due to possessing better mechanical properties, higher thermal resistance, recyclability and lighter weight [172]. The novel nanocomposites can be used to replace heavy metallic or non-critical parts such as dashboard, vent grills and truck beds in order to reduce the weight of the car. The advantages of reducing weight are a decrease in fuel consumption and less greenhouse gas emissions. Additionally, one key property that the nanocomposite must possess before it is suitable for automotive applications is the thermal stability, in that it must be able to withstand the high temperature of the engine or the heat involved during painting of the car [173]. Nanofillers or regular fillers such as talc can help increase the heat distortion temperature of polymers like PP. For instance, polyamide 6-clay hybrid nanocomposite, which is synthesized by the addition of nanoclay to polyamide-6 via in situ intercalation polymerization, has a higher heat distortion temperature when compared to pure polyamide-6. This can be achieved with as little of 5 wt% clay loading [174].

Another characteristic that is vital is enhanced mechanical properties and dimensional stability. The reason nanocomposites are used for this reason is that the nanofillers can be easily dispersed across the polymer matrix due to the high aspect ratio, which gives rise to composites with high tensile strength and modulus [173]. For example, Toyota Motor Corporation had successfully created a polyamide 6/clay polymer nanocomposite to be used in timing belt covers of cars [175]. This polymer/clay composite has better mechanical properties when compared to polyamide 6, with a Young Modulus’s of 2.1 GPa compared to the 1.1 GPa of polyamide 6 for a nanocomposite with 1.6 vol% of clay incorporated into it. The noticeable improvement in the mechanical properties is due to the good dispersion of clay filler in the polymer matrix and the strong ionic bond between polyamide 6 molecules and the silicate layer of the clay. The reason why this characteristic is important is because mechanical strength and dimensional stability are essential factors in manufacturing of automotive vehicles.

Additionally, polymer nanocomposites that are able to conduct electricity can be used in the electrical industry to be used in electronic components such as light-emitting diodes (LED), capacitors and actuators. This is possible as the addition of CNT as nanofiller in a conducting polymer resulted in a conducting nanocomposite with an increased capacitance. For example, the SWCNT/polyaniline (PANI) composite showed a high capacitance value of 310 F/g, which was higher than that of pure PANI due to the availability of increased active sites for Faradaic reactions [176]. Additionally, the capacitance of a MWCNT/polypyrrole (PPy) composite film exhibited a capacitance of up to 192 F/g, which is close to that of conducting polymers (around 220 F/g) [177]. The high capacitance and conductivity are attributed to several factors such as the increased surface area of the porous nanocomposite structure which provides excellent electrolyte access and the well-dispersed conductive MWCNT within the polymer matrix. The MWCNT matrices also provide a porous scaffold where a thin layer of PPy is electrodeposited; this greatly increases ionic transfer of the composite. Another study showed that SWCNT/PPy composite managed to reach a maximum of 265 F/g capacitance [178]. Thus, these polymer nanocomposites can be used as electrodes to create a supercapacitor.

Besides the automotive and electrical industry, non-toxic polymer composites can be applied in the biomedical field. One type of polymer nanocomposite that can be used in the medicinal filed is graphene/polymer nanocomposites, as most research has shown that graphene is biocompatible with human cells and has low cytotoxicity [179]. Other studies have reported that functionalized graphene is less toxic than its non-functionalized counterpart [180]. The large surface area (around 2600 m^2^g^−1^) [181] presented by the graphene is also useful for biomedical applications as it increases cellular interactions with molecules such as deoxyribonucleic acid (DNA) and ribonucleic acid (RNA). Graphene-based composites are used as biosensors that can accurately and sensitively detect the presence of molecules that might signal an impending disease, such as glucose, uric acid, heavy metals, cholesterol, and many others [182]. For example, Lian et al. [183] synthesized a graphene/chitosan composite to be used as molecularly imprinted membranes in an electrochemical sensor to detect uric acid.

Additionally, graphene-based nanocomposites can also be used in tissue engineering as scaffold for bone tissue due to their biocompatibility. For instance, Kim et al. [184] developed a GO/calcium carbonate (CaCO_3_) nanocomposite film that supported high viability of osteoblast cells and has high in vitro bone bioactivity. The compatibility with osteoblast cells is shown when the GO/CaCO_3_ composite showed higher apatite formation in simulated body fluid, as compared to pure graphene or GO. Sayyar et al. [185] also synthesized a graphene/polycaprolactone polymer composite that can be used in tissue engineering and drug delivery. The composite is biocompatible with human cells, conductive, has improved mechanical properties, and excellent processability.

Carbon nanoparticles are also broadly used in actuators. This is because the inclusion of carbon nanofillers such as CNT into conductive polymers managed to enhance its electrical and mechanical properties; which can be used to prepare low voltage actuators. These actuators are able to produce a larger force when compared to actuators created using the original polymers. This is shown when Mottaghitalab et al. [186] produced a CNT/PANI composite fiber with 2 wt% SWCNT loading that exerted an actuation strain of up to 2.2%, which is twice the amount produced from neat PANI.

Furthermore, CNTs/epoxy resin nanocomposite can be used as a mechanically enhanced composite that has many application fields. Epoxy resin is selected as a matrix for polymer composites due to its excellent electrical, mechanical and thermal characteristics, chemical resistance and stability. The epoxy composites are used in aerospace engineering or the aircraft sector due to its excellent thermal resistance and high specific strength (strength to weight ratio). These composites are used in the wing-tip fairings of the Lockheed Martin F-35. The CNT reinforced epoxy composites are also used in Tomahawk missiles and aircrafts. One example of an aircraft that contains epoxy/CNT composite is the Bell Boeing V-22 Osprey, where the nanocomposite consists of around 65% of its total mass [187]. The strength of this nanocomposite is proven when tensile tests have shown that an addition of 0.50 wt% of pure MWCNT into the epoxy polymer matrix will result in the increase of 45% in tensile strength and 77% in Young’s Modulus when compared to pure epoxy resin [188]. CNT/epoxy nanocomposites are also used in bullet-resistant vests, ultralight weight structures in the aerospace sector and sports equipment.

Polymer nanocomposites with carbon nanoparticles as nanofillers have also been known to be used in the fabrication of structural and electronic parts using 3D printing. This is due to the hopes that the carbon nanomaterials will impart enhancements to the mechanical or electrical properties to the materials used for 3D printing. One such example is the thermoplastic PU (TPU)/polylactic acid (PLA)/GO polymer nanocomposite developed by Chen et al. [189]. This composite was prepared via solution mixing and then extruded into filaments to be used for 3D printing using the fused filament fabrication (FFF) process. FFF begins with the filament passing through a moving and heated nozzle, where the melted material is then extruded and deposited onto the build plate one layer at a time to create a 3D structure [190]. The incorporation of 0.5 wt% of GO into the TPU/PLA polymer has resulted in an increase of 69.17% and 75.50% in yield point and tensile modulus, respectively. The reason for the enhancement in mechanical properties is due to the hydrogen bonding between the oxygen containing functional groups on the surface of GO with the urethane groups in TPU and carboxyl groups in PLA. Additionally, the TPU/PLA/GO nanocomposite could also be used as 3D printed scaffold for tissue engineering due to its low cytotoxicity to cell growth. The TPU/PLA composite with 0.5 wt% of GO supports the highest cell growth and proliferation when compared to 2 and 5 wt% loading of GO. Another example of a polymer composite that can be used for 3D printing is a functionalized graphene polymer (FGP) nanocomposite consisting of hydroxypropyl cellulose as the base polymer with graphene functionalized with magnetic iron (II, III) oxide (Fe_3_O_4_) as nanofillers [191]. This nanocomposite takes the form of an ink which is used to create 2D and 3D objects via extrusion-based printing process. The 3D printed FGP object could be used as a 3D grid for EMI shielding as the graphene and Fe_3_O_4_ both have EMI shielding properties [192]. Additionally, the presence of graphene in this nanocomposite also resulted in an electrically conductive material, with a conductivity of up to 580 Sm^−1^. This allows the 3D printed object to be applied in advanced electronics devices. The magnetic properties of this FGP composite also allows it to be used as an electro-magnetic device, such as a magnetic switch or capacitor, due to the magnetic properties of Fe_3_O_4_ [193]. Objects created using 3D printing could also come in a variety of forms such as pyramid, sphere, honeycomb or a grid, which further increases its application.

## 9. Conclusions

The research and development of carbon nanoparticles, many of which are derived from graphite, which are abundant and widely distributed across the globe (world reserves of natural graphite are estimated to be around 800 million tonnes) has led to the discovery and development of many novel polymer composites in the field of nanotechnology. Nevertheless, there are a few areas that need to be focused on in order to develop high performance polymer nanocomposites which incorporate the many unique and useful properties of the various carbon nanoparticles, such as high Young’s Modulus, tensile strength and electrical conductivity. One of which is improving the dispersion of the nanoparticles or nanofillers within the polymer matrix, as homogenous dispersion helps enhance their potential within the nanocomposites by decreasing inter-filler attraction which improves mechanical and electrical properties of the nanocomposite. Another area that has an opportunity to be further researched upon is the functionalization of carbon nanoparticles. This is because functionalization can help in the formation of strong interphase between the functionalized nanoparticles and the polymer matrix, which improves the mechanical properties and fracture behavior of the composite, while simultaneously enhancing the dispersion of the nanoparticles in the polymer matrix. For example, chemical bonding between carbon nanosheets and PS helped transfer stress across the polymer composite interface more efficiently when the composite is stretched. Another challenge is selecting the most suitable type of functionalization to be used on the carbon nanoparticles, as each of them have different effects.

Additionally, several polymer nanocomposite processing techniques were discussed here, such as in situ polymerization, solution mixing, and melt mixing. The selection of these preparation methods is important as it has an effect on the dispersion of the carbon nanoparticles and the interaction of the nanoparticles with the polymer matrix. Other components that need to be taken into account include the solubility of the polymer in the solution used, damage to the carbon nanoparticles caused by sonication and toxicity of solvents used. One of the processing techniques that needs further research is the melt blending technique, as it is the most compatible with the industrial practices of today. Additionally, alternative preparation routes should also be explored for the mass production of polymer nanocomposites in industry and to commercialize it in the market.

To reiterate, in order to develop high performance carbon nanoparticles based polymer composite, there are a few factors needed to be taken into account: the optimum functionalization method, a suitable polymer for the carbon nanoparticles to be dispersed into, the molecular or interface interaction between the polymer matrix and the carbon nanofillers, the nanocomposite processing conditions (temperature, sonication time and shear force). In conclusion, the key areas needed to be further developed and researched upon are the functionalization of carbon nanomaterials, dispersion of nanofillers with polymer matrix and interfacial interaction between the nanofillers and the matrix.

## Figures and Tables

**Table 1:** Comparison between various properties of different carbon nanoparticles. Data adapted from [46].

Carbon Nanoparticle	Dimension	Hybridization	Thermal Conductivity (Wm^−1^ K^−1^)	Electrical Conductivity (Sm^−1^)	Hardness
Graphene	2	sp^2^	4840–5300	Around 2 × 10^5^	Highest (for a single layer material)
Carbon nanotube (CNT)	1	Generally sp^2^	Around 3500	Depends on the CNT structure used	High
Graphene oxide	2	sp^3^	Around 72	0.1	High
Fullerene	0	Generally sp^2^	0.4	10^−8^	High

**Table 2:** Comparison of graphite oxide produced by Staudenmaier and Hummers’ method in terms of compositions. Data adapted from [66].

Method	Carbon (wt%)	Oxygen (wt%)	Water (wt%)	Ash (wt%)	Carbon to Oxygen Atomic Ratio
Hummers’	47.06	27.97	22.99	1.98	2.25
Staudenmaier	52.11	23.99	22.20	1.90	2.89

**Table 3:** Differences between premixed and diffusion flame.

Premixed Flames	Diffusion Flames
1. Many types of fuel can be used, as the premixed nature of the fuel and oxidizer promotes quick combustion, which results in an easily controllable flame that can be used widely in laboratories and industries.	Only fuels that need less air for complete combustion can be used.
2. The stoichiometry of the fuel mixture can be easily altered by manipulating the fuel to oxidizer ratio. Thus, the temperature of the flame can be changed.	The stoichiometry of the fuel mixture cannot be changed, and the temperatures of the flame cannot be controlled.
3. Premixed flame produces less soot than diffusion flame which will not hinder the production of the nanotubes.	Diffusion flames have a tendency to burn slowly and produce more soot. Thus, the fuel stream needs to be diluted with inert gas to prevent the soot from enveloping the metal catalysts [83].
4. There is a possibility that explosions can occur.	Diffusion flame is safer than premixed flame as it does not have such issues.
5. It is not limited by diffusion. Thus, a uniform reactive gas profile can be created using the burner system [84].	Mixing of fuel and gases is diffusion limited.

**Table 4:** Summary and comparison between CNT synthesis processes.

Method	Yield Rate	Single-Wall Carbon Nanotube (SWCNT) or Multi-Wall Carbon Nanotube (MWCNT)	Advantages	Disadvantages
Electric arc discharge	<75%	Both SWCNT and MWCNT.	- Can create nanotubes with little structural defects due to the high temperature used. - Simple and inexpensive process. - Can produce a large amount of nanotubes.	- The chirality of CNTs cannot be controlled. - The created nanotubes need to be purified if metal catalysts are used. - High temperature required for this method.
Laser ablation	<75%	Only SWCNT.	- High purity of SWCNT is able to be achieved. - The structure of SWCNT produced is better graphitized.	- Unable to produce nanotubes on a large scale. - High energy is needed for laser usage.
Chemical vapor deposition (CVD)	>75%	Both SWCNT and MWCNT.	- The highest yield out of the four synthesis methods. - High purity nanotubes are produced. - Fit for large-scale production. - Able to create nanotubes in many forms and structures.	- This method mainly favors the growth of MWCNT.
Flame synthesis method	<75%	Both SWCNT and MWCNT.	- It is a fast, simple and inexpensive process.	- This method has low catalyst yield. - Impurities were found on the crude nanotubes synthesized.

**Table 5:** Difference between covalent and non-covalent functionalization.

Covalent Functionalization	Non-Covalent Functionalization
Formation of stable, non-reversible covalent bonds.	Uses electromagnetic or adsorption forces including van der Waal forces, hydrogen bonds, π-π stacking and electrostatic force.
Change in hybridization from sp^2^ to sp^3^, and a loss of conjugation in carbon nanoparticles.	Electronic structure is maintained.
There is a change in electronic structure of the carbon nanoparticles.	No change in electronic structure of the carbon nanoparticles.
The particles are attached onto the sidewalls or on the tips of the carbon nanomaterials.	The particles are wrapped over the surface of the carbon nanomaterials.
Performed through amidation, esterification, halogenation, oxidation, and cycloaddition.	Performed through adsorption of polymers, surfactants and metal nanoparticles on the surface of the carbon nanoparticles.

**Table 6:** Various mechanical properties of carbon nanoparticles before and after functionalization.

Type of Carbon Nanoparticle	Type of Functionalization	Mechanical Property	Before Functionalization	After Functionalization	Ref.
Armchair SWCNT	Covalent functionalization	Buckling force	108 (nN/100 atoms)	88.6 (nN/100 atoms)	[100]
Graphite nanosheet/polystyrene (PS) nanocomposite	Supercritical water treatment	Tensile strength	9.06 MPa	27.23 MPa	[8]
Graphite nanosheet/PS nanocomposite	Supercritical water treatment	Impact strength	3.17 J/m	6.12 J/m	[8]
Carbon fiber/resin nanocomposite	Supercritical water treatment	Interfacial shear strength	52.8 MPa	58.2 MPa	[120]

**Table 7:** Various electrical properties of carbon nanoparticles before and after functionalization.

Type of Carbon Nanoparticle	Type of Functionalization	Electrical Property	Before Functionalization	After Functionalization	Ref.
MWCNT sheet	Acid functionalization	Electrical conductivity	236.37 × 10^2^ Sm^−1^	726.99 × 10^2^ Sm^−1^	[132]
MWCNT sheet	Acid functionalization	Electromagnetic interference (EMI) shielding effectiveness	50.15 dB	63.63 dB	[132]
SWCNT film	Doping using iodine monochloride (ICl) in dichloromethane (DCM) solvent	Reduction in electrical resistance	-	67% (normalized resistance is used rather than an absolute value)	[135]
SWCNT film	Doping using iodine monobromide (IBr) in DCM solvent	Reduction in electrical resistance	-	42% (normalized resistance is used rather than an absolute value)	[135]
Graphite nanosheet/Polystyrene (PS) nanocomposite	Supercritical water treatment	Electrical conductivity	10^−7^ Sm^−1^	25 Sm^−1^	[8]

**Table 8:** Dithiothreitol (DTT) decay rates of different carbon materials. Data adapted from [141].

Dithiothreitol (DTT) Decay Rates of Different Carbon Materials			
Carbon Nanoparticles		Soot	
Name	Decay Rate (pmol min^−1^ µg^−1^)	Name	Decay Rate (pmol min^−1^ µg^−1^)
Graphene	58.5 ± 6.6	Methane flame soot	33.6
Graphene oxide (GO)	160.7 ± 21.7	Propane flame soot	49 ± 7
SWCNT	38.9 ± 8.9	Hexane flame soot	27.0
SWCNT-OH	57.0 ± 7.2	Diesel soot	6.1
SWCNT-COOH	36.7 ± 0.2	Graphite	0.9

**Table 9:** Relative ratio of adenosine triphosphate (ATP) level of J774 cells after exposure to functionalized carbon nanoparticles. Data adapted from [141].

Carbon Nanoparticles	Relative Adenosine Triphosphate (ATP) Level of J774 Cells after Exposure
Graphene	0.67 ± 0.06
Graphene oxide (GO)	0.84 + 0.03
SWCNT	0.59 + 0.10
SWCNT-OH	0.93 + 0.01
SWCNT-COOH	0.88 + 0.02

**Table 10:** Properties of polymer nanocomposite before and after addition of functionalized carbon nanoparticles.

Polymer Matrix	Functionalized Carbon Nanofiller	Properties	Properties of Pure Polymer Matrix	Properties After Addition of Functionalized Carbon Nanoparticles	Ref.
Carbon fiber reinforced epoxy composite	0.5 wt% amine functionalized single-wall carbon nanotube (SWCNT)	Ultimate tensile strength	681 MPa	747 MPa	[146]
		Elastic modulus	62 GPa	74 GPa	
Poly(vinyl alcohol) (PVA)	3 wt% graphene functionalized with oxygen-containing functional groups	Tensile strength	65 MPa	110 MPa	[149]
		Young’s Modulus	2.1 GPa	4.8 GPa	
Epoxy	0.125 wt% graphene functionalized with oxygen-containing functional groups	Fracture toughness	1.03 MPa	1.70 MPa	[152]
		Fracture energy	325 Jm^−2^	700 Jm^−2^	
Polycarbonate (PC)	3 wt% graphene functionalized with oxygen-containing functional groups	Surface electrical resistance	8.8 × 10^13^ ohms	2.0 × 10^5^ ohms	[155]
Nafion composite	1 wt% tricyanohydro-fullerene, HC_60_(CN)_3_	Proton conductivity	0.16 Sm^−1^	0.34 Sm^−1^	[160]
Branched sulfonated poly(arylene ether ketone sulfone) (BSPAEKS)	0.75 wt% sulfonated fullerene	Proton conductivity	24.1 Sm^−1^	33.2 Sm^−1^	[161]

**Table 11:** Polymer nanocomposites prepared using in situ polymerization, solution mixing and melt blending.

Polymerization Method Used	Polymer Nanocomposite Product	Advantages of the Method	Disadvantages of the Method
1. In situ polymerization	Polystyrene (PS)/expanded graphite [162]	- The carbon nanofillers are easily dispersed within the polymer matrix. - The efficient dispersion results in stronger interactions between the nanofillers and the polymeric phase. - Used for nanocomposites that cannot be prepared using solution mixing or melt mixing.	- It is difficult to acquire materials that are suited to this process and costly equipment is needed.
	Polypyrrole (PPy)/Multi-walled Carbon Nanotube (MWCNT) [163]		
2. Solution mixing	Poly (methyl methacrylate) (PMMA)/expanded graphite [166]	- Result in nanocomposites with low percolation values.	- The use of large quantities of solvent may result in environmental damage. - High energy sonication is required for effective dispersion of nanofillers.
	Polyurethane (PU)/MWCNT [167]		
3. Melt blending	Polyamide-6/expanded graphite [168]	- A straightforward process that is relatively cheap. - Favored manufacturing technique of industrial practices.	- Agglomeration of nanofillers in polymer composites (poor dispersion). - Mechanical blending may damage the carbon fillers.
	High-density polyethylene (HDPE)/unmodified graphite [171]		

## Data Availability

Not applicable.

## Abbreviations

CNTs, carbon nanotubes; GO, graphene oxide; PS, polystyrene; SWCNT, single-wall carbon nanotube; MWCNT, multi-wall carbon nanotube; CVD, chemical vapor deposition; DWCNT, double-walled carbon nanotubes; C_60_, Buckminsterfullerene; MRI, magnetic resonance imaging; CT, computed tomography; g-C_3_N_4_, graphitic carbon nitride; KClO_3_, potassium chlorate; KMnO4, potassium permanganate; NaNO_3_, sodium nitrate; H_2_SO_4_, sulphuric acid, CO_2_, carbon dioxide; CO, carbon monoxide; PMMA, poly (methyl-methacrylate); RGO, reduced GO; SCF, supercritical fluid; XPS, X-ray photoelectron spectroscopy; HCl, hydrochloric acid; Ru, ruthenium; SPANI, sulfonated polyaniline; PS-NH_2_, amine-terminated polystyrene; NH_3_^+^, amine ions; COO^−^, carboxylate ions; ATR-FTIR, attenuated total reflection Fourier transform infrared; EMI, electromagnetic interference; DDQ, 2,3-dichloro-5,6-dicyano-1,4-benzoquinone; BAP, 1-bromoacetyl pyrene; Ce_2_O_3_, cerium(III) oxide; Al_2_O_3_, aluminum oxide; HRTEM, high-resolution transmission electron microscopy; IFSS, interfacial shear strength; ICl, iodine monochloride; IBr, iodine monobromide; DTT, dithiothreitol; ROS, reactive oxygen species; ATP, adenosine triphosphate; LDH, lactate dehydrogenase; FG, functionalized graphene; PVA, poly (vinyl alcohol); PP, polypropylene; HC_60_(CN)_3_, tricyanohydrofullerene; BPSPAEKS, branched sulfonated poly(arylene ether ketone sulfone); CTAB, cetyltrimethylammonium bromide; PU, polyurethane; DMF, dimethylformamide; LED, light-emitting diodes; PANI, polyaniline; PPy, polypyrrole; DNA, deoxyribonucleic acid; RNA, ribonucleic acid; CaCO_3_, calcium carbonate; PLA, polylactic acid; TPU, thermoplastic polyurethane; FFF, fused filament fabrication; FGP, functionalized graphene polymer; FE3O4, iron (II, III) oxide.
